# Self-reported functional and mobility limitations in mid-life: a systematic review of prevalence and association with chronic health conditions

**DOI:** 10.1186/s12889-026-28084-8

**Published:** 2026-06-19

**Authors:** Roee Hayek, Shmuel Springer, Rebecca T. Brown

**Affiliations:** 1 Faculty of Health Sciences, Department of Physical Therapy, The Neuromuscular & Human Performance Laboratory, Ariel University, Ariel, Israel; 2 Division of Geriatric Medicine, Perelman School of Medicine of the University of Pennsylvania, 3615 Chestnut Street, Philadelphia, Pennsylvania United States of America; 3 Geriatrics and Extended Care Program, Corporal Michael J. Crescenz VA Medical Center, Philadelphia, Pennsylvania United States of America; 4 Center for Health Evaluation Research and Promotion, Corporal Michael J. Crescenz VA Medical Center, Philadelphia, Pennsylvania United States of America; 5 Leonard Davis Institute of Health Economics, University of Pennsylvania, Philadelphia, Pennsylvania United States of America

**Keywords:** Middle-age, Mobility, Function, Early detection, Systematic review, Self-reported

## Abstract

**Background:**

Middle age is increasingly recognized as a critical period for the emergence of functional and mobility limitations. However, no prior systematic review has synthesized prevalence or incidence data specifically for this age group. This review aimed to summarize epidemiological evidence on self-reported functional and mobility limitations among middle-aged adults and to examine their associations with chronic conditions.

**Methods:**

This systematic review was registered in PROSPERO (CRD420251038883) and conducted in accordance with PRISMA 2020 guidelines. A search of PubMed, Scopus, Embase, and Web of Science was performed for studies published between 2000 and 2025. Eligible studies included population-based samples of midlife-related adults reporting prevalence or incidence of functional and/or mobility limitations. When data were available, associations between these limitations and chronic conditions were also examined. Methodological quality was assessed using the Joanna Briggs Institute checklist, and risk of bias was evaluated using the tool developed by Hoy et al. Evidence certainty was assessed using GRADE. Due to conceptual and methodological heterogeneity, a meta-analysis was not performed and findings were synthesized narratively.

**Results:**

Twenty-six population-based studies comprising over 500,000 participants from over 30 countries worldwide met the inclusion criteria. Reported prevalence of functional and mobility limitations varied widely by domain, instrument, and task. Across many studies, estimates fell within the range of 15% to 25%, while mobility limitation exceeded 40% in some settings. In studies assessing both domains, mobility limitations were generally reported more frequently than functional limitations. Chronic conditions were associated with functional and mobility limitations, particularly musculoskeletal pain and arthritis (Population-Attributable Risk [PAR]: 15.0%-38.3%), and multimorbidity (PAR: 12.0%-56.5%). In contrast, lower PAR estimates were reported for hypertension (3%). Most evidence was derived from cross-sectional studies, with heterogeneous age ranges and assessment tools, limiting comparability and causal inference.

**Conclusions:**

Functional and mobility limitations emerge during midlife worldwide and are associated with chronic conditions. The findings should be interpreted cautiously, given methodological variation across studies, including differences in measurement instruments, midlife age-group definitions, and outcome assessment, requiring standardization. Nevertheless, these findings may support earlier detection of functional and mobility changes and inform future prevention-oriented research and strategies.

**Trial registration:**

PROSPERO (CRD420251038883).

**Supplementary Information:**

The online version contains supplementary material available at https://doi.org/10.1186/s12889-026-28084-8.

## Introduction

The ability to move freely and carry out everyday tasks independently is a cornerstone of health and overall well-being [1]. However, aging is often accompanied by gradual changes in functional and mobility abilities [2, 3]. The World Health Organization defined healthy aging as “the process of developing and maintaining the functional ability that enables well-being in older age” [4], and therefore, it is essential to assess function and mobility to ensure autonomy and quality of life with age. These domains are frequently evaluated through self-report instruments, such as surveys and questionnaires, which typically cover basic activities of daily living (ADLs: bathing, dressing, transferring in or out of a bed or chair, toileting, feeding oneself, and walking across a room) and instrumental activities of daily living (IADLs: preparing a hot meal, shopping for groceries, making telephone calls, managing medications, doing work around the house, and managing money) [5, 6], as well as questions related to physical capacity, such as getting up from a chair, climbing stairs, and lifting and carrying objects [7].

Self-report instruments, which provide a practical and accessible means of capturing individuals’ perceived physical capacity and autonomy [8, 9], are frequently used by large-scale longitudinal studies to gather comprehensive data from diverse populations over time. They provide valuable insights into the patterns of functional and mobility competence, and how these are shaped by socio-demographic and health-related factors throughout the aging process [5–7, 10]. Understanding the onset and extent of functional and mobility limitations is critical for guiding timely interventions that preserve these abilities and support healthy aging [11].

Although “functional status” and “mobility” are often assessed or discussed in overlapping ways, particularly in studies evaluating intrinsic physical capacity, they represent distinct but related domains. While both rely on physical ability [12], functional status refers to a broader range of activities essential for independent living, such as preparing meals, maintaining personal hygiene, or managing household responsibilities [13]. The term “function”, however, is sometimes used more broadly in the literature to denote constructs such as physical or muscle function [14], which may create ambiguity about its meaning. When used in epidemiological research, self-reported functional assessments typically quantify limitations in ADLs and IADLs and thereby reflect functional status.

Mobility, on the other hand, is commonly defined as the ability to move independently from one place to another [14, 15]. It encompasses a broad spectrum of physical actions, including walking, climbing stairs, transitioning between postures, carrying objects, and safely navigating diverse environments, as well as movements such as kneeling, bending, reaching, and stooping, and even extends to more complex tasks such as exercising [1, 14–17]. The distinction between functional and mobility assessment domains is particularly important in terms of understanding and detecting changes in physical capacity across adulthood. The WHO International Classification of Functioning, Disability and Health (ICF) provides a standard framework for this distinction by classifying mobility as a domain that includes changing body position, lifting and carrying, and walking, whereas other activity domains, such as self-care and domestic life, encompass activities commonly assessed through ADL and IADL measures and capture key components of functional status [18].

Unlike functional assessment, which tends to involve tasks with relatively low mobility demands (e.g., walking across a room, transferring in or out of a bed or chair) and is therefore more susceptible to ceiling effects, mobility is often evaluated through higher-demand locomotor tasks and may therefore be detected earlier than functional limitations. Accordingly, mobility evaluation may offer a more sensitive lens for detecting subclinical declines in physical capacity as early as midlife [13, 14, 19].

Middle age represents a critical life course period during which subtle, progressive declines in function and mobility first emerge due to physiological changes such as slight decreases in muscle strength and neuromuscular coordination [20–22]. The onset of functional and mobility limitations often begins as early as age 45, with notable increases after age 50, as highlighted by Choi et al. [23] and Iezzoni et al. [24] who reported that 46% of mobility difficulties develop before age 50. Moreover, there is a well-established association between chronic health conditions, such as diabetes, cardiovascular disease, arthritis, and obesity, with declines in functional status and mobility among older adults [25, 26]. Given the high prevalence of these conditions in midlife [27, 28], it is important to understand their association with self-perceived physical capacity, as functional decline can occur even in the absence of chronic conditions, while some individuals with chronic conditions maintain their functional and mobility independence [29].

Although early signs of functional and mobility decline can emerge by midlife, this stage represents an important period for understanding the emergence of these limitations, given the established relevance of early recognition to population health. To our knowledge, no prior review has synthesized prevalence, incidence, and their associations with chronic conditions within a single framework focused on self-reported midlife limitations. This systematic review aims to address these gaps by synthesizing the existing evidence. Specifically, it examines (1) the prevalence and incidence of functional and mobility limitations among middle-aged adults based on self-reports, and (2) their association with chronic health conditions in middle-aged adults, as reported in studies that included relevant data.

## Methods

### Protocol and registration

The methodology for this systematic review was based on the Preferred Reporting Items for Systematic Reviews and Meta-Analyses (PRISMA) guidelines and conducted following the PRISMA 2020 checklist [30]. The complete PRISMA 2020 checklist is shown in Supplementary 1a and 1b. The corresponding review protocol is registered at PROSPERO (CRD420251038883). Before conducting the present review, a comprehensive search of PubMed, Scopus, Embase, and Web of Science, as well as the PROSPERO registration process (April 2025), was performed to verify that no similar systematic reviews addressing this topic were identified.

### Study selection

This review focused on original quantitative observational studies that reported descriptive estimates of prevalence and/or incidence of functional and/or mobility status limitations in middle-aged adults. To enhance conceptual clarity given the variability in definitions used across studies, outcomes were classified into two domains: Function, representing functional status and defined solely by assessments based on basic and instrumental activities of daily living (ADL/IADL) [14], and Mobility, which encompasses a broad spectrum of physical actions, ranging from walking and posture transitions to more complex tasks such as exercising. This operational distinction was also considered in relation to the WHO ICF activities and participation framework, particularly the distinction between mobility-related activities and self-care or domestic-life activities. Potentially overlapping tasks were considered in relation to task context and demand [18]. We considered both cross-sectional and longitudinal cohort studies conducted in the general population, where outcomes related to function and/or mobility were measured using self-reported instruments; studies meeting these criteria were eligible for inclusion. The age range selected for this review was 40–69 years, reflecting the predefined eligibility criteria and the variability in definitions of midlife across the literature [23, 24, 31–35].

### Search strategy

**Fig. 1:**
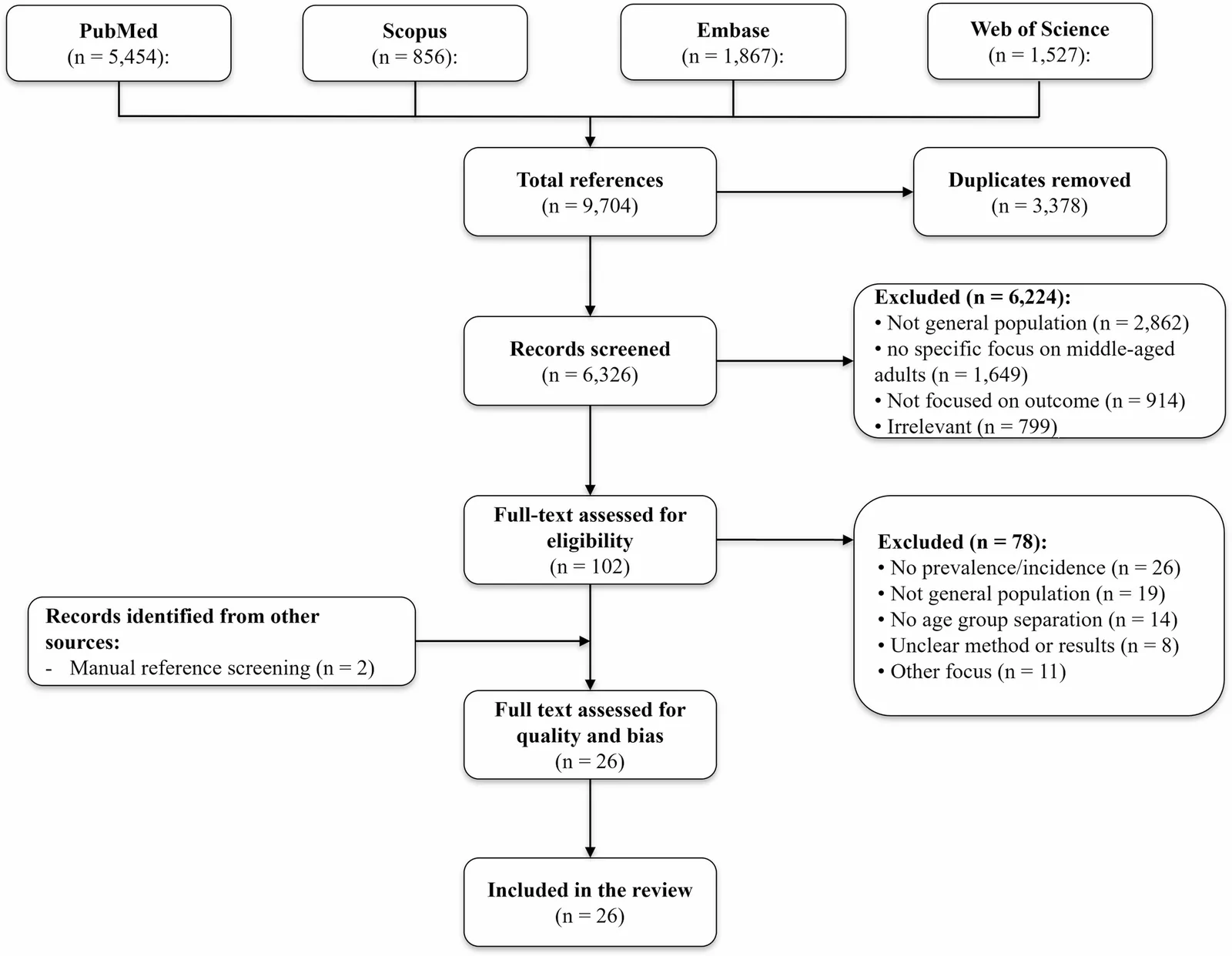
PRISMA flow diagram illustrating the article selection process

A comprehensive search was conducted in four electronic databases: PubMed, Scopus, Embase, and Web of Science, illustrated in Fig. 1. The search was performed between April 8 and April 10, 2026. The search was limited to peer-reviewed studies published in English between January 1, 2000, and March 31, 2026. The year 2000 was selected as the publication cut-off to focus on contemporary evidence relevant to current clinical and epidemiological practice, while still capturing publications from longitudinal cohorts initiated before 2000. The search strategy combined free-text keywords and controlled vocabulary terms (e.g., MeSH, Emtree, and database-specific thesauri) related to middle-aged populations and mobility, functional, and physical limitations or disabilities. The initial search was conducted broadly, focusing on population and outcome terms. To improve specificity, the search was subsequently refined by restricting terms to the title and abstract fields. Where necessary, additional epidemiological terms (e.g., “prevalence” OR “incidence”) were incorporated to further narrow the results. In cases of continued high retrieval volume, study design filters (e.g., “cohort”, “cross-sectional”, “wave” (representing repeated assessments in large longitudinal datasets)) were applied. Boolean operators (AND/OR/NOT) were used to combine the different search components for population, outcomes, and study design. The full and exact search strategies used for each database are provided in Supplementary 2a.

### Inclusion and exclusion criteria

The initial title and abstract screening were conducted by two reviewers (RH & SS), who applied the predefined inclusion and exclusion criteria to identify potentially relevant studies. Inclusion criteria included peer-reviewed, population-based studies (cross-sectional or longitudinal) that focused specifically on community-dwelling midlife-related adults aged 40–69 years. Studies were included if they reported clearly stratified results for this group, based on self-reported assessments of function (defined as ability to perform ADL or IADL tasks, even when not explicitly categorized as such) and/or mobility (encompassing a broad spectrum of physical actions, ranging from walking and posture transitions to more complex tasks such as exercising), with sufficient methodological detail to assess the outcomes of interest and ensure comparability. Specifically, sufficient detail was defined as including clear specification of the questions used to assess functional and mobility limitations, the response scales and classification criteria used to define these limitations, as well as their reported prevalence or incidence in the study population. These criteria reflect our focus on capturing the overall functional and mobility status of middle-aged populations to allow for consistent interpretation and inclusion in the review.

While the primary focus of this review was on functional and mobility limitations in midlife-related adults, several studies also reported associations with chronic conditions. When relevant data were available, these associations were analyzed as well.

Studies were excluded if they focused exclusively on older adults without providing information on middle-aged adults; did not specifically define a middle-aged group or clearly distinguish between middle-aged and older adults; or focused solely on individuals with specific medical conditions (e.g., serious illnesses, use of walking aids, neurologic disorders affecting motor, sensory, or cognitive function, or significant orthopedic injuries). In addition, non-English language publications, non-peer-reviewed publications, and studies with insufficient methodological clarity were also excluded. This selection strategy ensured relevance to the general middle-aged population while preserving methodological consistency across diverse sources.

### Chronic health conditions and functional and mobility limitations in midlife

Although not a primary inclusion criterion, several eligible studies examined the relationship between selected chronic health conditions and functional or mobility limitations using adjusted risk estimates, including odds ratios (ORs) [36], risk ratios (RRs) [37], hazard ratios (HRs) [38] and sub-hazard ratios (SHRs) [39], or Population-Attributable Risks (PARs) [36, 40], depending on study design and analytical approach. PAR quantifies the proportion of a given condition (here, functional and mobility limitations in middle-aged adults) attributable to specific exposure (here, chronic health conditions), provides insight into the overall burden of limitations, and identifies the conditions that contribute most significantly, highlighting opportunities for targeted prevention strategies [36]. These effect measures were synthesized descriptively; due to differences in study design, populations, outcomes, and analytical approaches, ORs, RRs, HRs, SHRs, and PARs are not directly comparable in magnitude, and no attempt was made to convert them to a common scale.

For studies reporting associations between chronic health conditions and functional and/or mobility outcomes, adjusted effect estimates were extracted, restricted to models accounting for sociodemographic characteristics, health-related behaviors, and coexisting conditions. From this subgroup of studies, the prevalence and incidence of chronic conditions among middle-aged adults were also extracted, where available, to provide clinical context. To ensure consistency, the term ‘association’ was used exclusively to refer to adjusted effect measures (OR, RR, HR, SHR, and PAR), relying solely on data synthesis rather than independent statistical recalculation.

Chronic health conditions were selected as a priori based on their high prevalence in midlife, frequent inclusion across the studies identified in this review and relevance to public health prevention and intervention strategies [41, 42]. Based on these criteria, the following conditions were examined when data were available: musculoskeletal pain and arthritis, overweight and obesity, diabetes, stroke, heart disease, hypertension, depression, and multimorbidity (defined as the presence of two or more chronic conditions).

### Critical appraisal, risk of bias and assessment of certainty of the evidence

All studies that underwent full-text screening were critically appraised for methodological quality and risk of bias using the Joanna Briggs Institute (JBI) Critical Appraisal Checklist for Studies Reporting Prevalence Data [43]. The JBI tool has been formally evaluated and is increasingly recommended for use in systematic reviews to assess prevalence data [44]. This tool assesses nine areas, including adequacy of sampling frame, sampling method, sample size, description of subjects and setting, coverage of identified sample, validity and reliability of condition measurement, adequacy of the statistical analysis, and management of response rates. Each item was scored ‘1’ if the answer was ‘Yes’ and ‘0’ if the answer was ‘No’ or ‘Unclear’. Based on the total score, studies were categorized as being of low (0–3 points), medium (4–6 points), or high (7–9 points) methodological quality. Critical appraisal scores were assigned based solely on the information available for the middle-aged population in each study and its specificity to the target population of this review. The final JBI scores are presented in Table 1, and full JBI scoring details are provided in Supplementary 3a.

**Table 1:** Overview of prevalence, data sources, and function and mobility assessment methods

Author and year of publication	Study design	Focus Country	Source, (years of data collection)	Sample size of middle-aged adults, (midlife age group)	Function and/or Mobility assessment method	Key findings (Prevalence/Incidence) (Mobility/function)
Iezzoni et al. (2001) [24] JBI^a^: Moderate (6) Risk of Bias^b^: Moderate (7)	Cross-Sectional study		NHIS-D (1994–1995)	6,593 (50–69)	Mobility: Assessment based on survey questions about difficulty walking a quarter mile, climbing 10 stairs, standing 20 min, and use of mobility aids. Scale categorized mobility difficulties into None, Minor: some difficulty or use of a cane/crutches. Moderate: a lot of difficulty or use of a walker. Major: completely unable or use of a wheelchair/scooter.	Prevalence: Mobility: • − 15.4% reported mobility difficulties: 6.4% with minor, 4.4% with moderate, and 4.6% with major difficulties • − 46.0% experienced onset before age 50
Martin et al. (2010) [42] JBI^a^: Moderate (6) Risk of Bias^b^: Low (8)	Repeated Cross-Sectional study		NHIS (11 consecutive annual cycles: 1997–2007)	5,500 to 7,400, depending on the survey year; independent nationally representative cross-sectional samples collected annually (50–64)	Function: Participants reported binary (yes/no) whether they had difficulty performing each of: Function: - ADLs - IADLs Mobility: - Physical Functions - Stooping, bending, kneeling, standing two hours, pushing/pulling large object, walking a quarter mile, climbing ten steps, sitting two hours, lifting and carrying 10 pounds, reaching over head and grasping small objects.	Prevalence: Function: • - ADL difficulties: – 1.1% in 1997 and 1.7% in 2007 • - IADL difficulties – 2.5% (remain stable with the years) Mobility: • − 42.0% had physical difficulties (remain stable)
Karvonen-Gutierrez & Ylitalo, (2013) [32] JBI^a^: Moderate (6) Risk of Bias^b^: Moderate (7)	Cross-sectional analysis nested within a cohort study		SWAN (2011)	376 - only women (55.9–67.7)	WHODAS 2.0 used to assesses difficulties: 5-point Likert scale, which ranges from no difficulty (0 points) to extreme self-reported difficulty (4 points) of 36 items assessing mobility and functional disability, score was categorized as: no problem (0%−4%); mild problem (5%−24%); moderate problem (25%−49%); severe problem (50%−95%); and extreme/complete problem (95%−100%).^+^	Prevalence: Function: • − 12.8% [9.40 to 16.14] had mild difficulties • − 6.1% [3.70 to 8.54] had moderate difficulties • − 4.3% [2.22 to 6.30] had severe difficulties Mobility: • − 26.3% [21.88 to 30.78] had mild difficulties • − 19.4% [15.41 to 23.41] had moderate difficulties • − 19.4% [15.41 to 23.41] had severe difficulties
Choi et al. (2016) [23] JBI^a^: Moderate (6) Risk of Bias^b^: Moderate (7)	Repeated Cross-Sectional study	USA	HRS (1998–2012): 8 biennial measurement waves (1998, 2000, 2002, 2004, 2006, 2008, 2010, 2012)	72,470 analyzed as a time series of independent cross-sections using cross-sectional weights (55–69)	Function: Participants reported binary responses (yes/no) of whether they had difficulty performing each of: Function: - ADLs - IADLs Mobility: - Physical ability measured with 9 activities: climbing a flight of stairs without resting; getting up from a chair; picking up a dime; reaching/extending arms up; pushing or pulling large objects; sitting for 2 h; lifting and carrying 10 pounds; stooping, kneeling, and crouching; and walking several blocks.	Prevalence: Function: • - ADL difficulties: – Baseline (1998): 12.0% – End-point (2012): 12.5% – Average: 12.5% (stable trend 0.02% [−0.77 to 0.80]). • - IADL difficulties: – Baseline (1998): 8.9% – End-point (2012): 11.2% – Average: 9.9% (increased by 1.33% [0.59 to 2.08] per year) Mobility: – - Baseline (1998): 56.8% – - Endpoint (2012): 55.7% – - Average: 57.9% (declined by 0.37% – [−0.63 to −0.10] per year)
Brown et al. (2017) [39] JBI^a^: High (9) Risk of Bias^b^: Low (8)	Prospective longitudinal cohort study		HRS (1992–2014): Biennial assessments for up to 20 years of follow-up.	6,874 community-dwelling adults (aged 50–56 at enrollment) with no prior functional impairment.	Function: Participants reported binary (yes/no) whether they had difficulty performing each of ADLs and IADLs	Incidence: Function: By the age of 64: • − 22% [21 to 23] developed new ADL impairment • − 19% [18 to 20] developed new IADL impairment
Brown et al. (2019) [41] JBI^a^: High (9) Risk of Bias^b^: Low (8)	Prospective longitudinal cohort study			5,540 (aged 50–56 at enrollment) with no prior functional impairment.		Incidence: Function: By the age of 64: • − 19.8% developed new ADL impairment • − 15.5% developed new IADL impairment
Tipirneni et al. (2021) [45] JBI^a^: High (7) Risk of Bias^b^: Low (9)	Repeated Cross-Sectional study		HRS (2002–2016): Comparison of two time points (2002 vs. 2016).	17,516 (55–64) Independent samples per wave.		Prevalence: Function: • - ADL difficulties: – 2002: 11.7% – 2016: 12.9% • - IADL limitations: – 2002: 8.9% – 2016: 10.1%
Okoro, (2018) [46] JBI^a^: High (7) Risk of Bias^b^: Low (9)	Cross-Sectional study		BRFSS-2016	174,413 (45–64)	Participants reported binary (yes/no) whether they had difficulty performing each of: - Mobility assessment - “Do you have serious difficulty walking or climbing stairs?” - Functional assessment- “Do you have difficulty dressing or bathing?”	Prevalence: Function: • Functional disability: 5.5% [5.3 to 5.7] Mobility: • Mobility limitations: 18.1% [17.7 to 18.5]
Bowling et al. (2019) [37] JBI^a^: High (9) Risk of Bias^b^: Low (9)	Cross-Sectional study		NHANES (2011–2016): pooled survey cycles	4,217 (50–64)	Limitations were assessed across four domains, where affirmative responses (“some difficulty” or greater) were classified as limitations: Mobility: 1. Functional (Physical) tasks: stooping, kneeling, lifting 10 lbs, standing, reaching, or grasping. 2. Mobility: walking ¼ mile, climbing 10 steps. Function: 3. ADLs 4. IADLs	Prevalence: Function: • - ADL difficulty: 15.0% [12.8 to 16.5] • - IADL difficulty: 17.0% [14.7 to 19.0] Mobility: • - Limitations in physical tasks: 34.0% [31.7 to 36.3] • − 11.0% [9.6 to 12.8] had mobility limitations
Heimbuch et al. (2023) [47] JBI^a^: High (8) Risk of Bias^b^: Moderate (7)	Repeated Cross-Sectional study		HRS (2006–2018): Biennial measurement waves	17,971 (50–64)	Function: Participants reported binary (yes/no) whether they had difficulty performing each of ADLs Weighted prevalence estimates were applied to account for the complex sampling design and adjusted to represent the U.S. non-institutionalized population, including age, sex, race/ethnicity, and non-response.	Prevalence: Function: - ADL difficulties (weighted prevalence): 2006: 12.5% [11.6 to 13.5] 2008: 11.5% [10.6 to 12.5] 2010: 13.0% [12.1 to 13.8] 2012: 11.9% [11.0 to 12.8] 2014: 12.6% [11.6 to 13.6] 2016: 12.4% [11.5 to 13.3] 2018: 12.7% [11.7 to 13.8] Trend: Fluctuating pattern with a slight overall increase across waves.
Griffith et al. (2017) [40] JBI^a^: High (7) Risk of Bias^b^: Low (10)	Cross-Sectional study	Canada	CCHS used an adapted ADL measure from the OMFAQ. (2008–2009)	15,799 (45–64)	Function: The OMFAQ contains 14 items pertaining to functional disability in basic ADLs and IADLs, answered on a six-point scale ranging from 1 (excellent functioning) to 6 (functioning totally impaired)	Prevalence: Function: • - ADL difficulties increase with age: – Ages 45–54: males 3.7% [2.3 to 5.0], females 8.9% [6.9 to 11.0] – Ages 55–64: males 5.6% [4.5 to 6.8], females 11.0% [9.3 to 12.7] • - IADL difficulties increase with age and are more common in females: – Ages 45–54: males 1.0% [0.5 to 1.5], females 3.3% [2.0 to 4.5] – Ages 55–64: males 2.6% [1.9 to 3.4], females 6.0% [4.8 to 7.2]
Melzer et al. (2005) [36] JBI^a^: High (7) Risk of Bias^b^: Moderate (7)	Cross-Sectional study		ELSA (2002)	11,392 (50–64)	Mobility: The assessment included self-reported difficulty walking 402 m, categorized as “no”, “some”, “much,” or “unable”.	Prevalence: Mobility: – - Women: 8% [7 to 9] – - Men: 9% [8 to 11]
Gardener et al. (2006) [48] JBI^a^: High (7) Risk of Bias^b^: Moderate (7)	Cross-Sectional study	UK	ELSA (2002)	11,216 (50–64)	The functional and mobility assessment included self-reported difficulty: Mobility: Walking 402 m, categorized as “no,” “some,” “much,” or “unable,” and difficulties with Function: ADLs from ELSA, determined by a “yes” or “no” response to specific ADL challenges.	Prevalence: Function: • ADL difficulties: – - Men: 16.0% (11% with 1–2 limitations and 5% with 3 or more limitations) and – - Women: 14% (10% with 1–2 limitations and 4% with 3 or more limitations) Mobility: – - Men: 18% [16 to 19] (9% with some difficulty, 5% with much difficulty, and 5% unable to walk a quarter mile) and – - Women: 19% [17 to 20] (9% with some difficulty, 5% with much difficulty, and 6% unable) reported mobility limitations
Burns et al. (2024) [49] JBI^a^: Moderate: (6) Risk of Bias^b^: Moderate (7)	Cross-Sectional study	Ten countries/regions: United States, India, China, Mexico, England, these total five regions were categorized as follows: Baltic States, Southern Europe, Eastern Europe, and Western Europe	Data of 2018, from six nationally datasets: United States (HRS), India (LASI), China (CHARLS), Mexico (MHAS), England (ELSA), and five regions of Europe (SHARE).	87,479 (50–64)	Mobility: LMFL was used as yes/no questions, including: walking 100 m/yards, rising from a chair, stooping/kneeling/crouching, and lifting/carrying 10 lbs/kilos.	Prevalence: Mobility: • - China: 40.6% (men), 58.7% (women) • - India: 51.0% (men), 66.2% (women) • - Mexico: 37.6% (men), 56.7% (women) • - United States: 52.2% (men), 63.0% (women) • - England: 37.8% (men), 48.3% (women) • - Baltic States: 39.1% (men), 55.0% (women) • - Scandinavia: 25.9% (men), 37.8% (women) • - Southern Europe: 35.1% (men), 48.6% (women) • - Eastern Europe: 37.4% (men), 51.3% (women) • - Western Europe: 34.8% (men), 47.4% (women)
Wang et al. (2021) [50] JBI^a^: High (8) Risk of Bias^b^: Low (8)	Repeated Cross-Sectional study	23 countries (America, Asia and Europe)	Harmonized population-based data from 6 major international cohorts (HRS, MHAS, ELSA, KLoSA, SHARE, CHARLS). The study utilizes a harmonized repeated cross-sectional design to evaluate decadal trends by comparing two distinct measurement windows: T1 (2004/05) and T2 (2014/15).	79,465 (55–65)	Function: Participants reported binary (yes/no) whether they had difficulty performing each of ADLs and IADLs	Prevalence: Function: • ADL global: – − 2004/05: Ranged from 1.5% (South Korea) to 16.7% (Poland) for women, and 2.4% (South Korea) to 17.1% (Poland) for men – − 2014/15: Ranged from 0.5% (South Korea) to 19.8% (China) for women, and 1.0% (South Korea) to 12.1% (China) for men • IADL global: – − 2004/05: Ranged from 2.2% (Czechia) to 9.2% (England) for women, and 0.4% (Greece) to 7.5% (England) for men. – − 2014/15: Ranged from 0.8% (South Korea) to 17.8% (China) for women, and 1.1% (Sweden) to 10.3% (Portugal) for men
Beller et al. (2023) [51] JBI^a^: High (7) Risk of Bias^b^: Low (9)	Repeated Cross-Sectional study	30 European countries.	European Social Survey, and GALI (2002–2018): collected biennially (2002, 2004, 2006, 2008, 2010, 2012, 2014, 2016, 2018) All samples are independent from each other from wave to wave.	167,902 (40–64)	Function: Activity limitations (None, Some, Severe) assessed with a single item, using the question: ‘Are you hampered in your daily activities in any way by any longstanding illness, disability, infirmity or mental health problem’? (‘yes, a lot’, ‘yes, to some extent’ or ‘no’).	Prevalence: Function: • Overall limitations: – − 2002 Range: 11.5% (Italy) to 38.4% (Czechia) – − 2018 Range: 10.2% (Italy) to 42.11% (Lithuania) – - Global Average: 25.5%. – - Minimum: 9.1% (Cyprus, 2006) – - Maximum: 49.3% (Ukraine, 2004) • Severe limitations: – − 2002 Range: 2.6% (Portugal) to 10.4% (Slovenia) – − 2018 Range: 1.5% (Italy) to 10.4% (Great Britain) – - Global Average: 5.8% – - Minimum: 1.1% (Italy, 2012) – - Maximum: 12.8% (Slovenia, 2006)
Picavet & Hoeymans, (2002) [52] JBI^a^: Moderate (6) Risk of Bias^b^: Low (8)	Repeated Cross-Sectional study	Netherlands	NetHIS (1990–1998): independent annual samples; a new representative group of the Dutch population was recruited for each of the 9 waves.	15,425 for all sample (45–64)	Measurement of physical disability was based on the OECD disability indicator: Mobility disability: Was defined as major difficulty or not able to carry 5 kg for 10 m and/or bending and picking something from the floor and/or walking 400 m. Function: ADL disability was defined as major difficulty or notable to do one of the following 10 activities of daily life: eating and drinking, getting in/out of a chair, getting in/out of bed, (un)dressing, transferring on one level, walking stairs, entering/leaving the house, transferring outdoors, washing face and hands and washing entirely.	Prevalence: (The report for middle-aged adults is based only on the data of NetHIS 1997–1998): Function: • ADL difficulties: 2.3% [1.8 to 2.8] Mobility: • Mobility difficulties: 8.5% [7.6 to 9.4]
Almazán-Isla et al. (2014) [31] JBI^a^: High (7) Risk of Bias^b^: Low (8)	Cross-Sectional study	Spain	WHODAS 2.0 (2008–2009)	604 (50–59, 60–69)	Function and Mobility: 5-point Likert scale, which ranges from no difficulty (0 points) to extreme self-reported difficulty (4 points) of 36 items assessing functional disability, score was categorized as: no problem (0%−4%); mild problem (5%−24%); moderate problem (25%−49%); severe problem (50%−95%); and extreme/complete problem (95%−100%).^+^	Prevalence: Function and Mobility: • 50–59: – - Mild disability: 20.1% – - Moderate disability: 10.3% – - Severe disability: 1.6% • 60–69: – - Mild disability: 25.5% – - Moderate disability: 8.1% – - Severe disability: 1.6%
Wilk et al. (2024) [53] JBI^a^: High (8) Risk of Bias^b^: Low (9)	Prospective longitudinal cohort study	Luxembourg	SHARE (2013–2020; Waves 5 (2013), 6 (2015), 7 (2017), 8 (2019–2020))	897 (baseline), 624 (wave 6), 628 (wave 7), 598 (wave 8); (50–64)	Function: Participants reported on a binary scale (yes/no) whether they had difficulty in performing ADLs and IADLs.	Prevalence: Function: ^#^ • Baseline (2013): 12.9% reported any limitation. • - Wave 6: 9.6% • - Wave 7: 10.2% • - Wave 8: 5.7%
Yonamine et al. (2016) [34] JBI^a^: High (8) Risk of Bias^b^: Low (9)	Cross-Sectional study	Brazil	VigiCardio Project, Cambé, Paraná (2011)	1,054 (40–49, 50–59, 60–69)	The SMAF used to assess difficulty in performing four activities: Function: getting up from the bed to an armchair or a chair, sitting and lying down; walking around the house. Mobility: Walking out of the house and going up and down stairs. Disability was considered a deficit in each function was graded from 0 (autonomy) to −3 (total help or dependence). The subjects were classified into three categories: no disability (score 0), mild disability (scores − 0.5, −1 and − 1.5) and moderate/severe disability (scores − 2 and − 3).	Prevalence: Function: • Get up, sit, lie down: – - Ages 40–49: mild: 4.8% moderate/severe: 0.2% – - Ages 50–59: mild: 10.4% moderate/severe: 0.3% – - Ages 60–69: mild: 13.8% moderate/severe: 2.1% • Walk around the house: – - Ages 40–49: mild: 0.6% moderate/severe: 0% – - Ages 50–59: mild: 3.0% moderate/severe: 0.3% – - Ages 60–69: mild: 1.3% moderate/severe: 1.7% Mobility: • Walk outside the house: – - Ages 40–49: mild: 1.9% moderate/severe: 0.4% – - Ages 50–59: mild: 5.5% moderate/severe: 1.6% – - Ages 60–69: mild: 7.2% moderate/severe: 1.3% • Go up/down stairs: – - Ages 40–49: mild: 12.4% moderate/severe: 0.8% – - Ages 50–59: mild: 20.8% moderate/severe: 2.5% – - Ages 60–69: mild: 33.3% moderate/severe: 2.5%
Boakye et al. (2024) [54] JBI^a^: Moderate (6) Risk of Bias^b^: Moderate (7)	Cross-Sectional study	Ghana	WHO SAGE Wave 2 (2014–2015)	458 (50–59), 680 (60–69)	Mobility: Mobility assessment method: Self-reported difficulty with mobility based on question: “Overall in the last 30 days, how much difficulty did you have with moving around?” Responses dichotomized into: • No difficulty • Difficulty (mild/moderate/severe/extreme)	Prevalence: Mobility: • − 50–59: 11.9% • − 60–69: 11.4%
Wilunda et al. (2015) [33] JBI^a^: Moderate (5) Risk of Bias^b^: Low (8)	Cross-Sectional study	Kenya	INDEPTH (2006–2007)	1,271 (50–59), 404 (60–69)	Mobility: Was assessed with the WHO and INDEPTH Questionnaire, using a five-point Likert scale: none vs. mild vs. moderate vs. severe vs. extreme/cannot do, of two questions: (1) how much difficulty did you have with moving around? (2) how much difficulty did you have in vigorous activities (such as cycling or working in the fields)? (where responses were either severe and extreme or cannot do, respondents were classified as having ‘poor’)	Prevalence: Mobility: • 50–59 (‘poor’ mobility): – - Men: 4.0% – - Women: 11.9% • 60–69 (‘poor’ mobility): – - Men: 4.2% – - Women: 17.5%
Ai et al. (2024) [38] JBI^a^: Moderate (6) Risk of Bias^b^: Moderate (7)	Prospective cohort study	China	CHARLS (2011–2018; waves: 2011, 2013, 2015, 2018)	6,241 (45–59), without ADL impairment at baseline	Function: Participants rated on a 4-point scale (no difficulty to unable to perform) whether they had difficulty in performing ADLs.	Incidence: Function: 772 out of 6,241 participants (12.4%) developed ADL impairment during follow-up (2011–2018)
Nagarkar et al. (2020) [55] JBI^a^: High (9) Risk of Bias^b^: Moderate (7)	Cross-Sectional study	India	Pune-FAAT (N/A)	553 (45–59)	Function and Mobility: Pune‑FAAT scale contained ten items: lifting (3–4 kg weight), bending, squatting, walking (1–2 km), climbing stairs, rising from chair and bed, bathing, using a toilet, dressing, and eating. The individuals were asked to rate on a four‑point Likert scale the level of difficulty they faced (no difficulty, some difficulty, most difficult, or cannot perform).	Prevalence: Function and Mobility: • Overall, 55.2% reported limitations. The prevalence increases significantly with age group: – - Ages 45–49: 40.3% – - Ages 50–54: 58.6% – - Ages 55–59: 69.8%
Peltzer et al. (2021) [56] JBI^a^: High (8) Risk of Bias^b^: Low (8)	Cross-Sectional study		LASI, Wave 1 (2017–2018)	34,098 (45–59)	Function: Participants reported on a binary scale (yes/no) whether they had difficulty in performing ADLs and IADLs.	Prevalence: Function: ADL difficulties: 9.9% IADL difficulties: 26.5% Combined ADL/IADL: − 29.3% had ≥ 1 limitation − 18.9% had ≥ 2 limitations
Zimmer et al. (2014) [35] JBI^a^: Moderate (6) Risk of Bias^b^: Low (9)	Prospective longitudinal cohort study	Taiwan	Survey of Health and Living Status of the Middle-Aged and Elderly in Taiwan (1996–2007; 4 waves: 1996, 1999, 2003, 2007).	1,344 (50–59 at the baseline), 1,555 (60–69 at the baseline).	Mobility: Participants reported on a binary scale (yes/no) whether they had difficulty in one of seven functions: 1. Raising hands over head 2. Grasping or turning objects with fingers 3. Lifting or carrying something weighing 11–12 kg such as two pecks of rice 4. Standing for 15 min 5. Squatting 6. Running a short distance such as 20–30 m 7. walking up two or three flights of stairs	Prevalence: * Mobility: Baseline limitation prevalence: – - Ages 50–59: 21.6% experienced at least one difficulty – - Ages 60–69: 38.0% experienced at least one difficulty

Values in square brackets indicate 95% confidence intervals (95% CIs) where reported in the original studies, for example [1.8 to 2.8]. Activities of daily living (ADLs: making phone calls, handling money, taking medication, shopping for groceries, and preparing a hot meals); Instrumental activities of daily living (IADLs: walking across a room, dressing, bathing, eating, transferring in/out bed, and toileting)

*NHIS-D* National Health Interview Survey on Disability, *LMFL* Large muscle functional limitation, *HRS *Health and Retirement Study, *SWAN* The Study of Women’s Health Across the Nation, *BRFSS* Behavioral Risk Factor Surveillance System data, *NHANES* National Health and Nutrition Examination Survey, *LASI* Longitudinal Aging Study in India, *CHARLS* China Health and Retirement Longitudinal Study, *MHAS* Mexican Health & Aging Study, *ELSA* English Longitudinal Study of Ageing, *SHARE* Survey of Health, Ageing, and Retirement in Europe, *NetHIS* Cross-sectional national health survey data, *WHODAS 2.0* World Health Organization Disability Assessment Schedule-2.0, *CCHS* Canadian Community Health Survey, *OMFAQ *Older Americans Resources and Services Multidimensional Functional Assessment Questionnaire, *Pune-FAAT* Pune Functional Ability Assessment Tool, *ESS* European Social Survey, *INDEPTH* International Network for the Demographic Evaluation of Populations and Their Health, *SMAF* Functional Autonomy Measurement System, *GALI* Global Activity Limitation Indicator

* - Only baseline point prevalence is reported. Subsequent analysis used trajectory modeling which does not yield aggregate prevalence or raw incidence rates for the follow-up period

+ - The functional assessment focused on two domains: getting around and self-care. Thus, prevalence estimates and associations are specific to these functions

# - Values represent the total limitations at each wave, including those already limited at baseline. Percentage decline is due to attrition

a - Superscript numbers indicate the final Joanna Briggs Institute (JBI) Critical Appraisal Score (0–9), agreed by both reviewers. Quality was rated “low” (≤ 3), “moderate” (4–6), or “high” (7–9)

b - Risk of bias was assessed using the modified Hoy et al. (2012) tool. Final ratings reflect consensus, with scores of 0–3 as ‘High’, 4–7 as ‘Moderate’, and 8–10 as ‘Low’

Risk of bias was assessed using the “Assessing risk of bias in prevalence studies: modification of an existing tool” instrument [57]. The tool consists of 10 items, with the first four related to external validity (targeting selection and non-response bias) and the remaining six assessing internal validity (focusing on measurement and analysis-related bias). Each item was rated either “yes” (with a value of 1, indicating a low risk of bias) or “no” (with a value of 0, indicating a high risk of bias). Items for which insufficient information was available were also scored ‘no’, as per the instrument’s original instructions. Importantly, the rating is inversely related to the risk: the higher the total number of “yes” responses, the lower the overall risk of bias in the study. In accordance with the Hoy et al. tool [57] the overall risk of bias for each study was determined by consensus between all reviewers. Studies were categorized as ‘High’ risk of bias (i.e., only 0–3 items received a “yes” response, reflecting substantial methodological limitations that may bias the reported prevalence estimates), ‘Moderate’ (4–7 items had a “yes” response), and ‘Low’ (8 or more items received a “yes” response, indicating minimal methodological limitations and limited potential for bias in prevalence estimation). Studies that were rated as having either moderate or low risk of bias were deemed eligible for data extraction and inclusion in the synthesis. Full risk of bias scoring details are provided in Supplementary 3b.

Certainty of the evidence was assessed using an adapted Grading of Recommendations Assessment Development and Evaluation (GRADE) approach for primary outcomes (prevalence and incidence of functional and mobility limitations) and secondary outcomes (associations with chronic conditions) [58–60]. The assessment considered four core GRADE domains: risk of bias, inconsistency, indirectness, and imprecision, together with other considerations, including potential publication or reporting bias. Certainty was rated as high, moderate, low, or very low. Because meta-analysis was not performed, judgments were based on the methodological profile of the included studies, the consistency and range of estimates, and the comparability of populations, outcome definitions, instruments, thresholds, and effect measures. In the absence of a single universally accepted prevalence-specific GRADE framework [44], outcome-level judgments were informed by general GRADE guidance [58, 60, 61] and by approaches used in recent prevalence-focused systematic reviews [62, 63]. The GRADE evidence profile is presented in Supplementary Table 4.

Two reviewers (RH & SS) assessed each study independently, with disagreements resolved through discussion. There were no disagreements between the reviewers regarding the eligibility of the included studies.

### Data extraction

Data extraction was conducted independently for the included studies by two reviewers (RH & SS). Documentation included study characteristics (authors, publication year, country, study design, and sample size), definitions of the target population, and participant age ranges (focused on the 40–69 mid-life group). The specific measurement tools used to assess functional and mobility limitations were extracted, as well as the prevalence and incidence of limitations alongside adjusted association estimates (OR, PR, HR, SHR, and PAR), where reported.

Although the review was designed to evaluate both prevalence and incidence, only three studies reported the latter. Consequently, while these data were included, the analysis focused on prevalence patterns.

Substantial methodological heterogeneity was identified, driven by disparate outcome definitions, varied assessment tools and scales, inconsistent classification thresholds for limitation, and divergent age stratification. Given that these variations reflect structural and conceptual heterogeneity rather than random variation, a meta-analysis was not conducted, as it was deemed inappropriate and would not yield valid summary results. Instead, a narrative synthesis was conducted to summarize patterns across populations and study designs.

## Results

A total of 9,704 records were identified through database searches (PubMed = 5,454; Scopus = 856; Embase = 1,867; Web of Science = 1,527), and 3,378 duplicates were removed (Fig. 1). After title and abstract screening of the remaining 6,326 records, 6,224 were excluded for not meeting the inclusion criteria (e.g., not general population, no specific focus on middle-aged adults, irrelevant outcome). A total of 102 full-text articles were assessed for eligibility. Of these, 78 were excluded for reasons such as lack of prevalence/incidence data [64], focus on non-general populations [65, 66], other study objectives, or inclusion of functional and mobility limitations within broader domains or broader disability constructs without being assessed in isolation [67], or lack of data stratification by age groups [68, 69]. Two additional records were identified through manual screening of the reference lists of included studies. Both were assessed for eligibility and included in the review; no additional manually identified records were excluded. This resulted in a total of 26 articles included in the final review (See Fig. 1).

All articles included in the synthesis were of moderate to high quality based on the JBI and risk of bias assessments (see Table 1), and no studies were excluded after full-text review because of these assessments. Certainty of evidence was moderate for prevalence estimates of functional and mobility limitations and low for incidence of functional limitations. Certainty was very low for associations of chronic conditions with functional and mobility limitations. Lower certainty ratings mainly reflected heterogeneity in definitions, instruments, thresholds, follow-up structures, and effect measures (see Supplementary Table 4).

The review included 26 relevant studies that assessed more than 500,000 middle-aged participants from multiple countries, including the United States (USA) [23, 24, 32, 37, 39, 41, 42, 45–47, 50], Canada [40], and United Kingdom (UK) [36, 48], as well as countries across Africa [33, 54], Asia [35, 38, 49, 50, 55, 56], Brazil [34], and Europe [31, 49–53]. Three studies examined populations from different countries [49–51].

A range of large databases were used across the included studies to assess functional and mobility limitations in middle-aged adults, including the National Health Interview Survey (NHIS) [24, 42], Health and Retirement Study (HRS) [23, 39, 41, 45, 47, 50], Behavioral Risk Factor Surveillance System (BRFSS) [46], National Health and Nutrition Examination Survey (NHANES) [37], Survey of Health, Ageing, and Retirement in Europe (SHARE) [49, 50, 53], Longitudinal Aging Study in India (LASI) [49, 56], China Health and Retirement Longitudinal Study (CHARLS) [38, 49, 50], Mexican Health & Aging Study (MHAS) [49, 50], and English Longitudinal Study of Ageing (ELSA) [36, 48–50].

The studies employed standardized assessments of mobility, including activities such as lifting or carrying weights [23, 35, 37, 42, 49, 52, 55], walking or running short distances [23, 24, 35–37, 42, 46, 48, 49, 52, 55], rising from a chair [23, 34, 49, 52, 55], and climbing stairs [23, 24, 34, 35, 37, 42, 46, 52, 55]. Most studies examined participants’ functional difficulties in performing one or more of six ADLs (bathing, dressing, transferring in or out of a bed or chair, toileting, feeding oneself, and walking across a room) and instrumental activities of daily living (IADLs: preparing a hot meal, shopping for groceries, making telephone calls, managing medications, doing work around the house, and managing money) [23, 37–40, 42, 45, 47, 48, 50, 53, 56].

Functional and mobility difficulties were categorized either as binary (yes/no) [23, 35, 39, 41, 42, 45–47, 49, 50, 53, 54, 56], or stratified by level of difficulty using three-point Likert scales [36, 37, 51], four-point Likert scales [24, 32, 38, 55] or five-point Likert scales [31, 33, 34]. Table 1 summarizes the datasets, methods used, and the prevalence and incidence of functional and mobility limitations.

Out of the 26 studies, 14 were cross-sectional studies [24, 31–34, 36, 37, 40, 46, 48, 49, 54–56], 7 were repeated cross-sectional studies involving independent samples across multiple waves [23, 42, 45, 47, 50–52], and 5 were longitudinal cohort studies [35, 38, 39, 41, 53]. Among the longitudinal designs, prevalence data were often restricted to baseline due to attrition or trajectory modeling, with only 3 studies reporting the incidence of new functional limitations [38, 39, 41].

Methodological heterogeneity was reflected in the assessment focus, with studies evaluating a single domain of function or mobility, and some addressing both. Specifically, eight studies reported separate prevalence estimates for both function and mobility [23, 32, 37, 42, 46, 48, 50, 52]; ten reported only function [38–41, 45, 47, 50, 51, 53, 56]; six reported only mobility [24, 33, 35, 36, 49, 54]; and two reported a combined measure, either in a single overall report [31] or using tools that assessed both function and mobility [55].

### Incidence of functional limitations

Among the three studies that reported incidence, two provided data from the United States [39, 41] and one from China [38]. Incidence data were reported only for functional limitations, with no studies reporting incidence of mobility limitations. Cumulative incidence estimates among U.S. middle-aged adults ranged from 19.8 to 22.0% for ADL difficulties and 15.5–19.0% for IADL difficulties [39, 41], with estimates reflecting the proportion of initially non-disabled individuals aged 50–56 at baseline who developed limitations by age 64 over the follow-up period. In a separate study based on a Chinese cohort, an cumulative incidence of 12.4% was reported among individuals aged 45–59 at baseline over a 7-year follow-up period (2011–2018) [38].

### Prevalence of functional and mobility limitations

A range of reported prevalences for functional and mobility limitations was observed, with most between 15.0% and 25.5% [24, 35, 39, 41, 48, 51, 53]; sample sizes in these studies ranged from 604 [31] to 167,902 participants [51]. For instance, Iezzoni et al. [24] reported that 15.4% of individuals aged 50–69 experienced mobility issues (*n* = 6,593), and Gardener et al. [48] observed that 16% of men aged 50–64 in England had functional limitations (*n* = 2,696).

Among the studies that assessed both function and mobility separately, a consistent internal disparity was observed, with mobility limitations showing consistently higher prevalence rates than functional limitations within the same study populations. This pattern remained evident across various age groups. For instance, in a sample aged 50–64, Martin et al. [42] reported a 42% prevalence for mobility limitations, which was markedly greater than the 1.7% for ADL and 2.5% for IADL difficulties. Another example was found by Choi et al. [23] which focused on a population aged 55–69, and reported a 57.9% prevalence of mobility limitations, compared to 12.5% for ADL limitations and 9.9% for IADL limitations (*n* = 72,470). This gap was also found by Okoro [46] among adults aged 45–64, where mobility limitations reached 18.1% compared to 5.5% for ADL limitations (*n* = 174,413).

The prevalence of functional limitations in middle-aged populations demonstrated marked variability across studies. Low prevalence rates, below 5%, were observed in several samples [34, 42, 50–52]. For instance, Picavet and Hoeymans [52], reported a 2.3% prevalence of ADL disability (*n* = 15,425), while Wang et al. [50] reported ADL prevalence as low as 0.5% in specific regions such as South Korea and IADL rates as low as 0.4% among men in Greece. The most common prevalence rates ranged between 10% and 20%. This range was identified in eight studies [23, 32, 34, 37, 45, 47, 48, 53] across different regions and populations. For example, in the US (10.1%−12.9%) [23, 45], the UK (14.0%−16.0%) [48], and other European countries [37, 53].

The prevalence of mobility limitations was relatively high among middle-aged populations. Prevalence rates below 5% were observed in only two studies, within specific population subgroups or for particular tasks. For example, among men in Kenya aged 50–59, the prevalence was 4.0%, whereas among Kenyan women in the same age group it was nearly three times higher [33]. Similarly, for specific tasks such as walking outside the house, prevalence ranged from 1.9% (ages 40–49) to 7.2% (ages 60–69), compared with 12.4%−33.3% for going up or down stairs in the same age groups [34]. Rates between 5% and 10% were similarly infrequent, including a prevalence of 8.5% reported among both men and women in the Netherlands [52] and 8.0% to 9.0% of men aged 50–64 in the UK, affecting an estimated 787,000 middle-aged adults in England [36]. In contrast, many studies identified mobility limitations in 15% to 20% of their populations, including in the US [24, 46], Taiwan [35], and England [48]. Prevalence rates exceeded 40% in four studies [23, 42, 49, 55], with the highest prevalence recorded by Nagarkar et al. [55], at 69.8% among Indian adults aged 55–59 (*n* = 553), based on an assessment tool that primarily evaluated mobility abilities. These high figures were further evidenced by Burns et al. [49] who found mobility limitations ranging from 35.0% to 66.0% across various global populations.

### Age-related variation in functional and mobility limitations across midlife

When focusing on studies restricted to midlife populations under age 65, the prevalence of functional limitations ranged from as low as 1.1% for ADL difficulties [42] to 17.0% [37]. Mobility limitations showed a broader range across studies and populations, with prevalence estimates ranging from 8.0% [36] to as high as 69.8% [55], including intermediate estimates of 18.1% [46], 42.0% [42], and 66.2% [49].

An age-related increase in the prevalence of functional and mobility limitations was evident across studies, with rates often doubling or tripling during the midlife period. For example, Nagarkar et al. [55] reported an increase from 40.3% at ages 45–49 to 69.8% at ages 55–59. Similarly, Yonamine et al. [34] reported mobility limitations for climbing stairs increasing from 12.4% at ages 40–49 to 20.8% at ages 50–59 and 33.3% at ages 60–69. Zimmer et al. [35] further documented this progression, finding that the prevalence of mobility limitations rose from 21.6% in adults aged 50–59 to 38.0% in those aged 60–69. However, this pattern was not uniform across all populations. Boakye et al. [54] reported relatively stable mobility limitation prevalence, with 11.9% among adults aged 50–59 and 11.4% among those aged 60–69. Similarly, data from Canada showed more modest differences across age groups, with ADL difficulties increasing from 3.7% to 5.6% at in men and from 8.9% to 11.0% in women between ages 45–54 and 55–64, suggesting a more gradual age-related pattern [40].

### Gender differences in functional and mobility limitations

Gender differences were reported across both functional and mobility outcomes, with higher prevalence generally reported among women than men. In Canada, among adults aged 45–64, women reported higher rates than men, with ADL difficulties at 3.7% versus 8.9% at ages 45–54 and 5.6% versus 11.0% at ages 55–64 [40]. In the cross-national study by Wang et al. [50] among adults aged 55–65, ADL prevalence in 2014/15 reached 19.8% in women and 12.1% in men, while IADL prevalence ranged up to 17.8% in women and 10.3% in men across participating countries. Mobility limitations also showed higher prevalence among women, for example in Kenya among adults aged 50–59 (11.9% in women vs. 4.0% in men) and 60–69 (17.5% vs. 4.2%) [33]. Additionally, in the multi-country analysis by Burns et al. [49] mobility limitation prevalence was higher in women than men among adults aged 50–64 in every region examined, including India (66.2% vs. 51.0%), China (58.7% vs. 40.6%), and the United States (63.0% vs. 52.2%).

### Sociodemographic and socioeconomic disparities in functional and mobility limitations

Several studies reported differences in functional and mobility limitations by race/ethnicity and socioeconomic indicators, although these variables were not consistently assessed across studies. In the United States, Okoro [46] reported higher mobility disability among Black adults (25.3%), Hispanic adults (21.8%), and American Indian/Alaska Native adults (33.3%) than among White adults (16.2%). Melzer et al. [36] found mobility disability of 23% in the poorest wealth quintile compared with 2% in the wealthiest quintile. In China, incident ADL impairment among adults aged 45–59 was higher in rural than urban participants (14.0% vs. 9.8%) and among participants classified as illiterate compared with those with primary education or above (20.4% vs. 9.6%) [38].

### Variation across regions and countries in functional and mobility limitations

Substantial variation in prevalence was observed across countries and regions. These comparisons are descriptive, as no formal statistical testing of differences between countries was reported. In a study of 30 European countries (ages 40–64), Beller et al. [51] reported an overall prevalence of 25.5%, with lower estimates observed in high-income countries such as Italy (10.2%) and Cyprus (9.1%), compared to higher estimates in countries such as Ukraine (49.3%) and Lithuania (42.1%). Similarly, in a multinational analysis of 23 countries, Wang et al. [50] reported lower ADL prevalence in countries such as South Korea (0.5%) and Sweden (1.1%), compared to higher estimates in China (up to 19.8%), with IADL prevalence ranging from 0.4% in Greece to 17.8% in China. For mobility limitations, Burns et al. [49] reported lower prevalence in high-income regions such as Scandinavia (25.9%), compared to higher estimates in countries such as India (66.2%) and Mexico (56.7%), with intermediate to relatively high values observed in the United States (52.2%−63.0%) and England (37.8%−48.3%). Consistent with these patterns, Nagarkar et al. [55] reported high overall prevalence (up to 69.8% at ages 55–59) in India, based on a multi-item functional and mobility assessment.

### Association of chronic conditions with functional or mobility limitations

Among middle-aged adults, 18.0–36.0% of the population had at least one chronic health condition [36, 37, 40], with multimorbidity present in 23.0–33.0% [37, 40]. Chronic conditions were associated with an increased risk of functional and mobility limitations. Multimorbidity and chronic musculoskeletal pain and arthritis were consistently associated with functional and mobility limitations across studies, with higher population-attributable estimates reported for these conditions (PAR: 12.0–56.5% and 15.0–38.0%, respectively) [36, 40]. Melzer et al. [36] reported lower PARs for stroke and hypertension at 2.0% and 3.0%, respectively. Longitudinal incidence data showed that arthritis, heart disease, and hypertension were associated with an increased risk of ADL impairment of 47%, 37%, and 21%, respectively [38]. ORs and PARs were reported for all conditions except obesity, while PRs and SHRs were reported for all conditions except multimorbidity and heart disease (for which PRs were reported, but SHRs were not). HRs were reported only for hypertension, heart disease, arthritis, and multimorbidity. Table 2 provides detailed data on chronic health conditions, including their prevalence, incidence, risk ratios, and PARs.

**Table 2:** Population frequency and adjusted risk estimates for chronic conditions associated with functional and mobility limitations

Condition	Population Frequency	Adjusted Risk Estimates of Chronic Conditions Associated with Functional and/or Mobility Limitations			
	Prevalence of the condition in the middle-aged population	Odds Ratio (OR) ^#^ OR (95% CI) of mobility limitations	Prevalence Ratio (PR) ^+^ PR (95% CI)	Hazard Ratios (HRs) ^–^ and Subhazard Ratios (SHRs) ^*^ HRs and SHRs (95% CI)	Population Attributable Risk (PAR) PAR (95% CI)
Chronic Musculoskeletal Pain and Arthritis	Griffith et al. (2017) [40]: 45–54 yrs – Male: 12.6% – Female: 16.1% 55–64 yrs – Male: 22.1% – Female: 36.2% Bowling et al. (2019) [37]: 37.1% Brown et al. (2017) [39]: 25.0% Brown et al. (2019) [41]: 25.6%	Melzer et al. (2005)[36]: • Arthritis: 1.71 (1.21–2.41) • Lower limb chronic pain: 6.17 (4.33–8.79) • Back pain: 2.58 (1.83–3.63)	Bowling et al. (2019) [37]: Function: • ADL limitations: 3.13 (2.28–4.28) • IADL limitations: 2.56 (1.89–3.48) Mobility: • Mobility limitations: 2.32 (1.58–3.38) • Physical limitations: 2.21 (1.86–2.63)	Function: HRs: Ai et al. (2024) [38]: • ADL limitations: 1.47 (1.26–1.71) SHRs: Brown et al. (2017) [39]: • ADL limitations: 2.02 (1.70–2.40) • IADL limitations: 1.58 (1.35–1.85)	Function: Griffith et al. (2017)^°^[40]: • ADL limitations: Not reported • IADL limitations: Males: 33.0% (−13.5–77.8) Females: 36.4% (17.6–53.9) Mobility: Melzer et al. (2005)^#^[36]: • Mobility limitations of: Arthritis: 15.0% (5.0–24.0) Chronic pain in lower limb: 38.0% (31.0–45.0) Back pain: 15.0% (9.0–20.0)
Obesity and overweight	Griffith et al. (2017) [40]: NR Bowling et al. (2019) [37]: – Obesity: 42.4% Brown et al. (2017) [39]: – Overweight: 42.0% – Obesity: 27.0% Brown et al. (2019) [41]: – Overweight: 42.3% – Obesity: 27.1%	Melzer et al. (2005) [36]: NR	Bowling et al. (2019) [37]: Function: • ADL limitations: 1.83 (1.43–2.35) • IADL limitations: 2.56 (1.89–3.48) Mobility: • Mobility limitations: 2.00 (1.56–2.57) • Physical limitations: 1.67 (1.43–1.94)	Function: HRs: Ai et al. (2024) [38]: NR SHRs: Brown et al. (2017) [39]: • ADL limitations: 1.69 (1.40–2.03) • IADL limitations: 1.28 (1.04–1.58)	NR
Diabetes	Griffith et al. (2017) [40]: 45–54 yrs – Male: 6.6% – Female: 6.6% 55–64 yrs – Male: 13.1% – Female: 8.7% Bowling et al. (2019) [37]: 16.4% Brown et al. (2017) [39]: 7.0% Brown et al. (2019) [41]: 7.4%	Melzer et al. (2005) [36]: 1.55 (0.99–2.43)	Bowling et al. (2019) [37]: Function: • ADL limitations: 1.24 (1.02–1.50) • IADL limitations: 1.39 (1.16–1.68) Mobility: • Mobility limitations: 1.18 (0.90–1.55) • Physical limitations: 1.10 (0.98–1.23)	Function: HRs: Ai et al. (2024) [38]: NR SHRs: Brown et al. (2017) [39]: • ADL limitations: 1.45 (1.17–1.78) • IADL limitations: 1.47 (1.13–1.90)	Function: Griffith et al. (2017)^°^[40]: • ADL limitations: 45–54: 5.7% (−6.9–17.9) 55–64: 10.0% (0.9–18.8) • IADL limitations: 19.0% (−13.1–60.0) Mobility: Melzer et al. (2005)^#^[36]: • Mobility limitations: 2.0% (1.0–4.0)
Stroke	Griffith et al. (2017) [40]: 45–54 yrs – Male: 0.9% – Female: 0.6% 55–64 yrs – Male: 1.7% – Female: 1.7% Bowling et al. (2019) [37]: 3.1% Brown et al. (2017) [39]: 2.0% Brown et al. (2019) [41]: 1.6%	Melzer et al. (2005) [36]: 2.36 (1.27–4.38)	Bowling et al. (2019) [37]: Function: • ADL limitations: 1.62 (1.19–2.20) • IADL limitations: 1.38 (1.04–1.84) Mobility: • Mobility limitations: 1.20 (0.76–1.87) • Physical limitations: 1.30 (1.10–1.54)	Function: HRs: Ai et al. (2024) [38]: NR SHRs: Brown et al. (2017) [39]: • ADL limitations: 2.25 (1.57–3.25) • IADL limitations: 2.25 (1.53–3.31)	Function: Griffith et al. (2017)^°^[40]: • NR Mobility: Melzer et al. (2005)^#^[36]: • Mobility limitations: 2.0% (1.0–4.0)
Heart Disease (coronary heart disease, heart failure, heart attack, angina)	Griffith et al. (2017) [40]: 45–54 yrs – Male: 7.0% – Female: 2.8% 55–64 yrs – Male: 12.2% – Female: 6.2% Bowling et al. (2019) [37]: – Heart failure: 2.1% – Coronary heart disease: 5.3% Brown et al. (2017) [39]: 7.0% Brown et al. (2019) [41]: 7.0%	Melzer et al. (2005) [36]: • Angina: 2.48 (1.65–3.73) • Heart attack: 1.88 (1.13–3.10)	Bowling et al. (2019) [37]: Function: • ADL limitations: 1.58 (0.94–2.65) • IADL limitations: 1.41 (0.90–2.23) Mobility: • Mobility limitations: 1.37 (0.79–2.39) • Physical limitations: 1.21 (1.01–1.44)	Function: HRs: Ai et al. (2024) [38]: • ADL limitations: 1.37 (1.09–1.71) SHRs: Brown et al. (2017) [39]: NR	Function: Griffith et al. (2017)^°^[40]: • ADL limitations: 45–54: 18.7% (5.4–32.4) 55–64: 14.5% (5.5–23.7) • IADL limitations: 45–54: 29.7% (−10.6–71.6) Mobility: Melzer et al. (2005)^#^[36]: • Mobility limitations: 3.0% (0–5.0)
Hypertension	Griffith et al. (2017) [40]: NR Bowling et al. (2019) [37: 61.2% Brown et al. (2017) [39]: 29.0% Brown et al. (2019) [41]: 28.8%	Melzer et al. (2005) [36]: 1.12 (0.82–1.53)	Bowling et al. (2019) [37]: Function: • ADL limitations: 1.65 (1.30–2.09) • IADL limitations: 1.51 (1.21–1.87) Mobility: • Mobility limitations: 1.48 (1.20–1.81) • Physical limitations: 1.34 (1.17–1.53)	Function: HRs: Ai et al. (2024) [38]: • ADL limitations: 1.21 (1.01–1.45) SHRs: Brown et al. (2017) [39]: • ADL limitations: 1.18 (1.02–1.37) • IADL limitations: 1.18 (1.00–1.39)	Function: Griffith et al. (2017)^°^[40]: • NR Mobility: Melzer et al. (2005)^#^[36]: • Mobility limitations: 3.0% (−5.0–10.0)
Depression	Griffith et al. (2017) [40]: 8.7% Bowling et al. (2019) [37]: 17.4% Brown et al. (2017) [39]: 16.0% Brown et al. (2019) [41]: 15.8%	Melzer et al. (2005) [36]: 2.03 (1.57–2.62)	Bowling et al. (2019) [37]: Function: • ADL limitations: 1.91 (1.58–2.32) • IADL limitations: 1.87 (1.50–2.32) Mobility: • Mobility limitations: 1.37 (0.97–1.93) • Physical limitations: 1.51 (1.37–1.67)	Function: HRs: NR SHRs: Brown et al. (2017) [39]: • ADL limitations: 1.43 (1.21–1.69) • IADL limitations: 1.48 (1.23–1.77)	Function: Griffith et al. (2017)^°^[40]: • ADL limitations: 45–54, women: 8.8% (1.8–15.7) 55–64, women: 8.7% (3.8–13.4) • IADL limitations: 45–54 yrs: Men: 26.3% (−5.1–58.5) Women: 20.9% (4.4–35.9) 55–64 yrs, women: 18.0% (10.3–25.7) Mobility: Melzer et al. (2005)^#^[36}: • NR
Multimorbidity^±^	Griffith et al. (2017) [40]: 45–54 yrs – Male: (1):28.7% (2+): 7.8% – Female: (1): 25.3% (2+): 11.7% 55–64 yrs – Male: (1): 31.0% (2+): 19.3% – Female: (1): 35.8% (2+): 20.6 Bowling et al. (2019) [37]: – (2): 23.3% – (3): 18.2% Brown et al. (2017) [39]: NR Brown et al. (2019) [41]: NR	Melzer et al. (2005) [36]: Compared to none: • Two conditions: 2.45 (1.24–4.84) • Three conditions 2.85 (1.15–7.08)	Bowling et al. (2019) [37]: NR	Function: HRs: Ai et al. (2024) [38]: – (1): 1.40 (1.16–2.59) – (≥ 2): 1.79 (1.48 − 2.15) SHRs: Brown et al. (2017) [39]: NR	Function: Griffith et al. (2017)^°^[40]: • The PAR for functional limitations varies based on the combination of conditions: 24.0% (7.8–39.7) to 56.5% (1.2–100.0) Mobility: Melzer et al. (2005)^#^[36]: • The PAR for mobility limitations varies based on the number of conditions: 12.0% (5.0–19.0) to 15.0% (5.0–24.0)

Activities of daily living (ADLs(; Instrumental activities of daily living (IADLs(; Confidence interval (CI); Not reported (NR)

# Analyses were adjusted for sex, social class, qualification, wealth, smoking status and alcohol intake

+ Analyses were adjusted for age, sex, race/ethnicity, education, and cigarette smoking and the other chronic conditions

*Analyses were adjusted for all listed variables and calculated by using competing risks regression to account for the competing risk for death

° Analyses were adjusted for living alone (yes, no), adjusted total household income to the provincial low-income cut-off (above median, below median), ethnicity (white, non-white), BMI (< 25, 25–30, > 30), other comorbid conditions (any of Alzheimer’s disease, Parkinson’s disease or stroke)

– Analyses were adjusted for age at the follow-up in 2018, region, gender, place of residence, marriage, education, smoking, alcohol consumption, socialization, pain, falls

± In the context of multimorbidity prevalence and incidence in the middle-aged population, the number inside the brackets indicates the number of conditions

### Chronic musculoskeletal pain and arthritis

Chronic musculoskeletal pain and arthritis affected 12.6–37.1% of middle-aged adults [24, 36, 37, 40]. These conditions were consistently associated with functional and mobility limitations and showed notable attributable risks among the chronic conditions examined in the included studies. ORs for mobility limitations were up to 6.2 [36], and PRs up to 3.1 [37], while SHRs for functional limitations were up to 2.0 [39]. PARs for functional and mobility difficulties ranged from 15.0 to 38.0% [24, 36, 40]. The HR for incident ADL impairment associated with arthritis was 1.47 [38].

### Overweight and obesity

Overweight (BMI 25–29.9) and obesity (BMI ≥ 30) were prevalent in 27.1%−42.4% of middle-aged adults [37, 39]. Obesity was associated with PRs of 1.8 and 2.6 [37] and SHRs of 1.7 and 1.3 [39], for ADL and IADL limitations, respectively. ORs and PARs were not reported by the included studies.

### Diabetes

Diabetes was found in 7.4–16.4% of the middle-aged population [40, 41]. Having diabetes was associated with an OR of 1.5 for mobility limitations [36], and a PR of 1.1 to 1.4 for functional limitations [37]. Brown et al. [39] reported a SHR of 1.5 for incident ADL impairment. Diabetes also contributed to PARs of 3.0% for mobility limitations and up to 19.0% for functional limitations [36], increasing to 29.6–34.6% when combined with other conditions, such as heart disease or arthritis [40].

### Stroke

Stroke was relatively rare in middle-aged adults (0.6–3.1% prevalence) [37, 40]. The effect measures for functional and mobility disability following a stroke included an OR of 2.36 for mobility limitations [36], PRs of 1.2 to 1.6 for functional difficulties [37], and a SHR of 2.2 for functional difficulties [39]. However, the PAR reported by in Melzer et al. [36] for mobility limitations was relatively low, at 2.0%.

### Heart disease

Heart disease affected 7.0–9.0% of middle-aged adults overall [37, 39–41] and showed age-related increases in prevalence, from 7.0% in men and 2.8% in women ages 45–54 to 12.2% and 6.9%, respectively, at ages 55–64 [40]. Heart conditions, including ischemic heart disease, angina, heart attack, and heart failure, significantly impacted function and mobility in midlife. Individuals who had angina or had experienced a heart attack had ORs of 2.5 and 1.9 for mobility limitations, respectively [36], while individuals with heart failure had PRs ranging from 1.2 to 1.6 for mobility limitations [37]. Additionally, ischemic heart disease contributed to PARs of up to 29.7% for functional limitations in middle-aged men [40]. Heart disease was associated with an increased risk of incident ADL impairment (HR = 1.37).

### Hypertension

The prevalence of hypertension was 28.8–61.2% among middle-aged adults [37, 39]. Hypertension was associated with an OR of 1.1 for mobility limitations [36]. Functional difficulties were linked to PRs ranging from 1.3 to 1.6 [37] and a SHR of 1.2 [39]. Additionally, hypertension had a relatively low PAR of 3.0% for mobility difficulties [36], and was associated with an HR of 1.21 for incident ADL impairment [38].

### Depression

The prevalence of depression ranged from 8.7% [40] to 17.4% [37] among middle-aged adults. Depression was associated with both functional and mobility limitations. Melzer et al. [36] reported an OR of 2.03 for mobility limitations. Bowling et al. [37] reported PRs ranging from 1.37 for mobility limitations to 1.91 for ADL limitations. Brown et al. [39] reported SHRs of 1.43 for ADL limitations and 1.48 for IADL limitations. Griffith et al. [40] reported PARs for depression-related functional limitations in selected age-sex strata. For ADL limitations, PARs among women aged 45–54 and 55–64 were 8.8% and 8.7%, respectively. For IADL limitations, PARs reached 26.3% among men aged 45–54, 20.9% among women aged 45–54, and 18.0% among women aged 55–64. PARs for mobility limitations were not reported.

### Multimorbidity

Multiple conditions were common, with 23.3% of participants having two conditions, 18.2% three, 14.5% four, and 16.4% five or more [37]. Wilk et al. [53] reported that middle-aged adults with multimorbidity were 3 times more likely to experience limitations compared to those with one or no conditions. ORs for functional and mobility limitation increased progressively from 2.4 for two conditions to 2.8 for three or more conditions [36], with PARs varying according to the combinations of conditions, reaching as high as 56.5% [40]. The risk of incident ADL impairment increased with the number of chronic conditions (HR = 1.37–1.73) [38].

## Discussion

To our knowledge, this is the first systematic review to specifically integrate evidence on the prevalence and incidence of self-reported functional and mobility limitations and their associations with chronic conditions in middle-aged adults across diverse settings. This area has gained increasing recognition in recent years, with accumulating evidence underscoring its public health relevance. The findings of this review highlight the high prevalence of functional and mobility limitations in midlife, supporting the potential value of earlier recognition of such limitations in this age group.

The relatively limited number of eligible studies reveals a notable research gap and underscores the need for more standardized, age-specific research in midlife. While the primary objective was to evaluate both prevalence and incidence, most of the included studies were cross-sectional, and only five longitudinal cohort studies were identified [35, 39, 41, 53]. Among these, only three reported incidence. Ai et al. [38] reported that 12.4% of adults aged 45–59 developed ADL impairment over 7 years, whereas Brown et al. [39, 41] reported that 15.5%−22.0% of initially non-disabled adults aged 50–56 developed ADL or IADL impairment by age 64. This scarcity of longitudinal data limits the ability to delineate early progression patterns in this age group, highlighting the need for further research on incidence of these conditions in mid-life.

### Midlife age range and age-group variation in functional and mobility limitations

The inclusion of only 26 studies necessitates caution in interpreting the findings, as they may not fully capture the heterogeneity of the middle-aged population or the complexity of functional and mobility decline. A central source of heterogeneity lies in the varying definition of “middle age”. While typically defined as 45–64 years, three studies included participants up to age 69 [23, 24, 32], and four studies provided stratified data for both 50–59 and 60–69 groups [31, 33–35].

Data for participants aged 60–69 were included in this review. Including individuals in late midlife allowed us to examine the continuity of age-related changes into later life, when functional and mobility limitations become more pronounced, thereby strengthening the interpretation of age-related trajectories beginning in midlife. As global life expectancy continues to rise and the gap between total and functional life expectancy widens [70], gains in longevity are outpacing gains in years lived without functional limitation, suggesting that the chronological boundaries of middle age may require reassessment or alternative stratification. Yet heterogeneity in existing literature underscores the need for greater consistency and precision in defining midlife to improve comparability and interpretation.

Including participants after age 65 may yield higher prevalence estimates and therefore warrants cautious interpretation; however, substantial limitations in functional status and mobility are already evident earlier in midlife. Estimates restricted to midlife populations under age 65 show that functional limitations reached 17.0% [37]. Notably, the highest prevalence of mobility limitations across the included studies in this review was observed before age 65. For example, Burns et al. [49] reported mobility limitation prevalence ranging from 25.9% in Scandinavia to 66.2% in India among adults aged 50–64, with similarly high estimates observed in countries such as the United States and England. The highest prevalence was reported by Nagarkar et al. [55], reaching 69.8% before age 60.

While Zimmer et al. [35] reported an almost two-fold increase in mobility limitations, from 21.6% at ages 50–59 to 38.0% at ages 60–69, additional studies reported more modest increases. For example, Wilunda et al. [33] reported prevalence ranging from 11.9% (50–59 years) to 17.5% (60–69 years) in women, while Boakye et al. [54] found similar levels across age groups (11.9% among those 50–59 years vs. 11.4% among those 60–69 years). Yonamine et al. [34] showed that prevalence was higher among individuals aged 60–69 compared with those aged 40–49 and 50–59; however, differences were task-dependent. For instance, stair-climbing difficulties increased from 12.4% at ages 40–49 to 33.3% at ages 60–69, whereas indoor walking did not follow this pattern, with lower prevalence at ages 60–69 (1.3%) than at ages 50–59 (3.0%). Notably, although Choi et al. [23] reported a mobility limitation prevalence of 57.9% among adults aged 55–69, the mean age was 61 years, suggesting that these changes are already occurring during late midlife.

### Functional and mobility limitations

The variation observed across different tasks underscore a fundamental lack of standardized definitions of “mobility” and “function”, leading to conceptual overlap and inconsistent use of physical capacity measures across domains [14]. This ambiguity may lead to lower prevalence estimates in midlife, as preserved performance in basic ADL and IADL tasks can mask emerging limitations in more demanding activities, such as getting up from a chair. To enhance clarity, outcomes were classified into two domains: Function and Mobility, with function defined using ADL and IADL, reflecting the measurement approaches used in most of the studies included in this review, which supports this distinction [23, 37–42, 45, 47, 48, 50, 53, 56]. The ICF provides a useful conceptual anchor for this distinction. Within its activities and participation component, mobility captures movement-related activities such as changing body position, transferring, lifting and carrying, walking, and moving around, whereas other activity domains, including self-care and domestic life, encompass daily living activities commonly assessed through ADL/IADL-based functional measures [18]. The distinction between function and mobility may offer a structured basis for interpreting findings and may serve as a practical approach for future assessments.

The practical impact of this terminological ambiguity is reflected in the divergent prevalence rates reported for functional and mobility limitations in midlife. Functional limitation prevalence varies widely across studies, with some estimates falling below 5% and reaching as low as 0.4%−0.5% in certain regional samples [34, 42, 50–52], while the most common estimates range between 10% and 20% [23, 32, 34, 37, 45, 48, 53]. In contrast, mobility limitations are generally higher and more widely distributed, frequently exceeding 40% and reaching 69.8% in samples using mobility-focused instruments [7, 35, 49]. These differences may reflect variation in measurement approaches and potential ceiling effects. Their consistent direction within studies assessing both domains further suggests that mobility items may reflect higher task demands and capture limitations not reflected by many ADL/IADL-based functional measures.

Although methodological variation across studies limits direct comparison of prevalence estimates between independent samples, studies that assessed both function and mobility within the same population allow a more comparable evaluation. In these within-study comparisons, mobility limitations are consistently more prevalent than functional limitations, as demonstrated by Martin et al. [42] (42% vs. 1.7%) and Bowling et al. [37] (34% vs. 15%). Such evidence supports distinguishing function and mobility as separate assessment domains, as mobility-based measures capture limitations not reflected in ADL and IADL assessments, which were developed for older populations and are more prone to ceiling effects in midlife [71–73].

Functional and mobility limitations in this review are based on self-reported data, which are susceptible to recall bias, social desirability, and cultural variation in the perception of limitation [74]. Interpretation is further constrained by heterogeneous definitions and measurement formats, as binary classifications and Likert-type scales may yield different prevalence estimates [75]. Definitions of ‘limitation’ varied across studies, ranging from any reported difficulty to complete inability to perform a task. Such methodological variability contributes to non-uniform prevalence estimates and limits comparability across studies. Importantly, no consistent pattern was evident between higher prevalence estimates and assessed risk of bias or methodological quality, suggesting that variability is more likely driven by methodological and population differences rather than systematic bias. Given the substantial heterogeneity across measurement scales, outcome domains, and age strata, a formal meta-analysis was not conducted, as pooling across such heterogeneous studies would have provided limited interpretability and limited added value.

To address these limitations, the use of assessment tools specifically calibrated for midlife is warranted. One such instrument is the Mobility in Middle-Age Questionnaire (MMQ), developed by an international team of experts, which integrates current mobility capacity with perceived one-year change and demonstrates substantially lower ceiling effects (3.9%) compared with traditional measures such as the PF-10 (34.5%) [76].

### Gender differences in functional and mobility limitations

Higher functional and mobility prevalence rates among females compared with males were observed across diverse geographic settings and population-based samples included in this review [33, 40, 49, 50]. While the present review did not examine causal mechanisms, the greater burden observed among women may partly reflect changes associated with the menopausal transition [77]. This transition has been linked to declines in physical performance and is consistent with epidemiological evidence of higher disability prevalence among female than male [78, 79]. These findings underscore the importance of heightened attention to functional and mobility health among women during midlife.

### Sociodemographic and socioeconomic disparities in functional and mobility limitations

Several included studies reported variation in functional and mobility limitations by race/ethnicity and socioeconomic status [36, 38, 46]. These findings suggest that functional and mobility limitations in midlife may not be distributed equally across population subgroups. However, these indicators were not consistently reported or analyzed across studies, limiting systematic synthesis of disparities. Future research should more consistently report sociodemographic and socioeconomic stratification to better capture disparities in functional and mobility limitations during midlife.

### Variation across regions and countries in functional and mobility limitations

Regional variation suggests that national income does not necessarily translate into better functional health. However, these comparisons should be interpreted as descriptive, as statistical differences between countries were not formally assessed. Substantial variation was observed across countries within the same analyses, with lower prevalence reported in several high-income settings, including Italy (10.2%) and Cyprus (9.1%), alongside higher estimates in countries such as Ukraine (49.3%) and Lithuania (42.1%) [51]. Similarly, Wang et al. [50] reported that ADL prevalence ranged from 0.5% to 1.1% in high-income countries such as South Korea and Sweden, compared with up to 19.8% in China. Importantly, they further reported relatively higher estimates in countries such as Poland and Portugal. Furthermore, a relatively high prevalence of mobility limitations was observed in high-income countries, including the United States (52.2%−63.0%) and England (37.8%−48.3%), with estimates comparable to, or in some cases exceeding, those reported in India (51.0%−66.2%) and Mexico (37.6%−56.7%) [49]. The reason for the relatively high prevalence observed in some high-income countries requires further study; however, Burns et al. [49] suggested that socioeconomic inequality and comorbidity burden may contribute to these patterns, while differences in measurement tools may also affect comparability [51].

Repeated cross-sectional studies suggest that trends over time may differ across countries. In the United States, mobility limitations declined by 0.37% annually between 1998 and 2012 [23]. Similar decreases were observed between 2002 and 2018 in Italy (from 11.52% to 10.24%) and Switzerland (from 20.02% to 19.27%). In contrast, France experienced an upward trend, with prevalence increasing from 25.06% in 2006 to 29.28% in 2018 [51]. As statistical significance of these changes was not formally assessed, the reported differences reflect descriptive changes over time rather than confirmed temporal trends and should therefore be interpreted cautiously. Nevertheless, the observed shifts highlight the evolving nature of functional and mobility limitations as a global public health concern requiring continued attention to screening and intervention.

### Association of chronic health conditions

Chronic health conditions represent a considerable burden in middle age and are closely associated with functional and mobility limitations. The chronic conditions included in this review were selected based on their high prevalence in midlife populations and their established evidence for impacting functional health [41, 42]. To ensure methodological rigor, adjusted effect estimates (ORs, PRs, HRs, SHRs, and PARs) were extracted only from studies accounting for sociodemographic characteristics, health-related behaviors, and coexisting conditions. Yet methodological variation between studies, including differences in effect measures (ORs, PRs, HRs, SHRs, and PARs), limits direct comparison of magnitude; thus, the findings represent a descriptive synthesis and warrant cautious interpretation. For this reason, differences between conditions should be interpreted as descriptive patterns rather than as a hierarchy of association strength, particularly when estimates come from different studies, outcome definitions, or effect measures. This is also relevant for PAR estimates, which reflect population-level contribution rather than association strength alone.

Multimorbidity was consistently associated with functional and mobility decline in midlife, a concerning finding given that 23.3% of middle-aged adults report two conditions and 16.4% five or more [36, 37, 40]. Within studies using the same analytic framework, condition-specific patterns were more interpretable. In Melzer et al. [36] using the same ELSA sample and mobility outcome, lower-limb chronic pain showed the highest mobility estimates (OR 6.17; PAR 38.0%), whereas stroke and hypertension showed lower estimates (ORs 2.36 and 1.12; PARs 2.0% and 3.0%, respectively). A similar pattern was observed in Griffith et al. [40] where higher PARs for functional limitations were reported for arthritis, reaching 36.4% for IADL disability and up to 56.5% for selected multimorbidity combinations, while lower single-condition PARs were reported for diabetes and heart disease in several age-sex strata. In Brown et al. [39], incident functional impairment also showed higher SHRs for arthritis (ADL 2.02; IADL 1.58) than for hypertension (ADL 1.18; IADL 1.18). with diabetes, depression, and obesity generally falling between these estimates. These findings should be interpreted within the context of the specific measures and study designs used, and as descriptive within-study patterns rather than direct rankings across included studies.

Importantly, obesity, which represents a major public health factor with 42.4% prevalence in this review [37, 39], and prevalence ratios ranging from 1.67 to 2.56 for mobility limitation [37], remains unquantified at the population level due to a lack of PAR data. Additional research is needed to address this gap and determine total population impact attributable to obesity.

Interestingly, Bowling et al. [37] found that PRs associated with chronic conditions were higher for functional (2.56–3.13) than for mobility outcomes (2.21–2.32), even though mobility-related limitations were more prevalent. This pattern should be interpreted cautiously. It may indicate that functional and mobility limitations are related but represent distinct domains, with potentially different relationships to chronic health conditions and different patterns of development over time. However, longitudinal studies are needed to clarify the temporal relationship between chronic disease burden and the development of functional and mobility limitations in midlife. It should be acknowledged that functional and mobility limitations can develop independently of the presence of chronic conditions and may even occur in their absence [29]. Conversely, chronic disease may be present without evident limitations. This underscores the importance of monitoring function and mobility alongside disease status and supports a shift from a disease-centered model of healthy aging toward one that places function and mobility at its core [29].

### Implications for aging health and future directions

The findings of this review underscore the importance of early identification of functional and mobility limitations in midlife and monitoring of chronic disease burden. Midlife may represent a relevant stage for tracking changes in functional health that could inform prevention-oriented public health strategies. Given that the synthesized findings rely largely on self-reported epidemiological measures, their application in primary care or community surveillance should be further evaluated. Such work should use validated, midlife-specific tools and examine their value for identification and monitoring [76]. The potential value of early identification for supporting healthier aging also depends on greater consideration of effective prevention-oriented strategies and interventions for midlife populations, and on evaluating whether these tools can capture meaningful change over time.

This review was restricted to self-reported limitations; however, self-reported and performance-based measures are not interchangeable and may provide complementary information on functional health [80, 81]. Combining perceived limitations with objective performance-based measures that are sensitive to identifying functional and mobility limitations in middle-aged adults may support a more comprehensive assessment [82, 83], although the optimal approach for midlife populations requires further evaluation.

Future research should prioritize the use of standardized measures calibrated for use in midlife to improve comparability across studies, as well as clearer age definitions. In addition, more longitudinal studies are needed to better characterize the incidence and progression of functional and mobility decline over time.

### Limitations

This review has several limitations that should be considered when interpreting the findings. Most included studies were cross-sectional, with relatively few longitudinal data available, limiting conclusions regarding progression over time. Considerable heterogeneity was observed across studies in the definitions and measurement of functional and mobility limitations, as well as in age ranges, which in some cases extended beyond 65 years, reducing comparability and contributing to lower certainty of evidence for incidence and especially for associations with chronic conditions. These limitations further highlight the need for standardized outcome definitions, comparable effect measures, and longitudinal research. Additionally, The reliance on self-reported data may introduce recall, reporting, and cultural biases [74]. Finally, the search was restricted to English-language, peer-reviewed studies, which may have introduced language and publication bias. This restriction may also have limited the representation of evidence from non-English-speaking regions, some of which were included in this review.

## Conclusions

In this systematic review, we found that functional and mobility limitations are prevalent during midlife. Prevalence estimates were reported for both functional and mobility limitations across diverse populations and settings. In studies that assessed both domains separately within the same populations, higher prevalence of mobility limitations was consistently observed, likely reflecting differences in task demands and potential ceiling effects inherent to functional measures. Chronic conditions, particularly multimorbidity and musculoskeletal disorders, contribute to these limitations, reinforcing the close link between disease burden and functional health in midlife. These findings highlight midlife as an important period for identifying and monitoring functional and mobility limitations. Addressing modifiable risk factors and improving assessment tools tailored to midlife may support earlier identification of functional and mobility changes and help inform prevention-oriented strategies. 

## Supplementary Information

Supplementary Material 1.

## Data Availability

The datasets used and/or analysed during the current study are available from the corresponding author on reasonable request.
