# Supplementary material for: Self-reported functional and mobility limitations in mid-life: a systematic review of prevalence and association with chronic health conditions
Source: BMC Public Health. 2026 Jun 19;26:3244. doi: 10.1186/s12889-026-28084-8 (PMC13741494; doi:10.1186/s12889-026-28084-8)
Supplement: Supplementary file 1 [file 12889_2026_28084_MOESM1_ESM.docx]

**Supplementary**

**Supplementary 1a: PRISMA 2020 for Abstracts Checklist**

| **Section and Topic** | **Item #** | **Checklist item** | **Reported (Yes/No)** |
| --- | --- | --- | --- |
| **TITLE** | | |  |
| Title | 1 | Identify the report as a systematic review. | Yes |
| **BACKGROUND** | | |  |
| Objectives | 2 | Provide an explicit statement of the main objective(s) or question(s) the review addresses. | Yes |
| **METHODS** | | |  |
| Eligibility criteria | 3 | Specify the inclusion and exclusion criteria for the review. | Yes |
| Information sources | 4 | Specify the information sources (e.g. databases, registers) used to identify studies and the date when each was last searched. | Yes |
| Risk of bias | 5 | Specify the methods used to assess risk of bias in the included studies. | Yes |
| Synthesis of results | 6 | Specify the methods used to present and synthesise results. | Yes |
| **RESULTS** | | |  |
| Included studies | 7 | Give the total number of included studies and participants and summarise relevant characteristics of studies. | Yes |
| Synthesis of results | 8 | Present results for main outcomes, preferably indicating the number of included studies and participants for each. If meta-analysis was done, report the summary estimate and confidence/credible interval. If comparing groups, indicate the direction of the effect (i.e. which group is favoured). | Yes |
| **DISCUSSION** | | |  |
| Limitations of evidence | 9 | Provide a brief summary of the limitations of the evidence included in the review (e.g. study risk of bias, inconsistency and imprecision). | Yes |
| Interpretation | 10 | Provide a general interpretation of the results and important implications. | Yes |
| **OTHER** | | |  |
| Funding | 11 | Specify the primary source of funding for the review. | Yes |
| Registration | 12 | Provide the register name and registration number. | Yes |

**Supplementary 1b: PRISMA 2020 Checklist**

| **Section and Topic** | **Item #** | **Checklist item** | **Location where item is reported** |
| --- | --- | --- | --- |
| **TITLE** | | |  |
| Title | 1 | Identify the report as a systematic review. | Title page |
| **ABSTRACT** | | |  |
| Abstract | 2 | See the PRISMA 2020 for Abstracts checklist. | Supplementary data |
| **INTRODUCTION** | | |  |
| Rationale | 3 | Describe the rationale for the review in the context of existing knowledge. | Introduction - Background section |
| Objectives | 4 | Provide an explicit statement of the objective(s) or question(s) the review addresses. | Introduction - Objectives |
| **METHODS** | | |  |
| Eligibility criteria | 5 | Specify the inclusion and exclusion criteria for the review and how studies were grouped for the syntheses. | Inclusion and Exclusion Criteria section |
| Information sources | 6 | Specify all databases, registers, websites, organisations, reference lists and other sources searched or consulted to identify studies. Specify the date when each source was last searched or consulted. | Search Strategy |
| Search strategy | 7 | Present the full search strategies for all databases, registers and websites, including any filters and limits used. | Supplementary data |
| Selection process | 8 | Specify the methods used to decide whether a study met the inclusion criteria of the review, including how many reviewers screened each record and each report retrieved, whether they worked independently, and if applicable, details of automation tools used in the process. | Study selection |
| Data collection process | 9 | Specify the methods used to collect data from reports, including how many reviewers collected data from each report, whether they worked independently, any processes for obtaining or confirming data from study investigators, and if applicable, details of automation tools used in the process. | Data Extraction |
| Data items | 10a | List and define all outcomes for which data were sought. Specify whether all results that were compatible with each outcome domain in each study were sought (e.g. for all measures, time points, analyses), and if not, the methods used to decide which results to collect. | Data Extraction |
|  | 10b | List and define all other variables for which data were sought (e.g. participant and intervention characteristics, funding sources). Describe any assumptions made about any missing or unclear information. | Data Extraction |
| Study risk of bias assessment | 11 | Specify the methods used to assess risk of bias in the included studies, including details of the tool(s) used, how many reviewers assessed each study and whether they worked independently, and if applicable, details of automation tools used in the process. | Critical Appraisal - JBI tool |
| Effect measures | 12 | Specify for each outcome the effect measure(s) (e.g. risk ratio, mean difference) used in the synthesis or presentation of results. | ORs, PRs, HRs, SHRs, PARs specified |
| Synthesis methods | 13a | Describe the processes used to decide which studies were eligible for each synthesis (e.g. tabulating the study intervention characteristics and comparing against the planned groups for each synthesis (item #5)). | Critical Appraisal and Data Extraction |
|  | 13b | Describe any methods required to prepare the data for presentation or synthesis, such as handling of missing summary statistics, or data conversions. | Data Extraction |
|  | 13c | Describe any methods used to tabulate or visually display results of individual studies and syntheses. | Results, Tables |
|  | 13d | Describe any methods used to synthesize results and provide a rationale for the choice(s). If meta-analysis was performed, describe the model(s), method(s) to identify the presence and extent of statistical heterogeneity, and software package(s) used. | Data Extraction |
|  | 13e | Describe any methods used to explore possible causes of heterogeneity among study results (e.g. subgroup analysis, meta-regression). | Data Extraction |
|  | 13f | Describe any sensitivity analyses conducted to assess robustness of the synthesized results. | Not applicable |
| Reporting bias assessment | 14 | Describe any methods used to assess risk of bias due to missing results in a synthesis (arising from reporting biases). | Not applicable |
| Certainty assessment | 15 | Describe any methods used to assess certainty (or confidence) in the body of evidence for an outcome. | Methods; Results; Supplementary Table 4 |
| **RESULTS** | | |  |
| Study selection | 16a | Describe the results of the search and selection process, from the number of records identified in the search to the number of studies included in the review, ideally using a flow diagram. | Results, Figure 1. |
|  | 16b | Cite studies that might appear to meet the inclusion criteria, but which were excluded, and explain why they were excluded. | Results |
| Study characteristics | 17 | Cite each included study and present its characteristics. | Results, Table 1 |
| Risk of bias in studies | 18 | Present assessments of risk of bias for each included study. | Results, Table 1, Supplementary |
| Results of individual studies | 19 | For all outcomes, present, for each study: (a) summary statistics for each group (where appropriate) and (b) an effect estimate and its precision (e.g. confidence/credible interval), ideally using structured tables or plots. | Results, Tables |
| Results of syntheses | 20a | For each synthesis, briefly summarise the characteristics and risk of bias among contributing studies. | Results, Table 1 |
|  | 20b | Present results of all statistical syntheses conducted. If meta-analysis was done, present for each the summary estimate and its precision (e.g. confidence/credible interval) and measures of statistical heterogeneity. If comparing groups, describe the direction of the effect. | Results, Tables |
|  | 20c | Present results of all investigations of possible causes of heterogeneity among study results. | Results, Tables |
|  | 20d | Present results of all sensitivity analyses conducted to assess the robustness of the synthesized results. | Not applicable |
| Reporting biases | 21 | Present assessments of risk of bias due to missing results (arising from reporting biases) for each synthesis assessed. | Not applicable |
| Certainty of evidence | 22 | Present assessments of certainty (or confidence) in the body of evidence for each outcome assessed. | Supplementary Table 4 |
| **DISCUSSION** | | |  |
| Discussion | 23a | Provide a general interpretation of the results in the context of other evidence. | Discussion |
|  | 23b | Discuss any limitations of the evidence included in the review. | Discussion |
|  | 23c | Discuss any limitations of the review processes used. | Discussion |
|  | 23d | Discuss implications of the results for practice, policy, and future research. | Discussion |
| **OTHER INFORMATION** | | |  |
| Registration and protocol | 24a | Provide registration information for the review, including register name and registration number, or state that the review was not registered. | Protocol and registration |
|  | 24b | Indicate where the review protocol can be accessed, or state that a protocol was not prepared. | Protocol and registration |
|  | 24c | Describe and explain any amendments to information provided at registration or in the protocol. | Protocol and registration |
| Support | 25 | Describe sources of financial or non-financial support for the review, and the role of the funders or sponsors in the review. | Described |
| Competing interests | 26 | Declare any competing interests of review authors. | Declared |
| Availability of data, code and other materials | 27 | Report which of the following are publicly available and where they can be found: template data collection forms; data extracted from included studies; data used for all analyses; analytic code; any other materials used in the review. | Methods, Tables, Supplementary data |

**Supplementary 2a: Search Strategy for Systematic Review**

The following are the final search strategies used in each database.

**PubMed**

The initial broad search yielded 38,839 results using the following query:

("Middle Aged"[MeSH] OR "middle aged adults" OR "midlife" OR "mid-life" OR "middle-aged adults" OR "middle-aged individuals" OR "45-64" OR "55-64" OR "60-69") AND ("mobility limitation" OR "mobility impairments" OR "mobility disability" OR "functional impairments" OR "functional disability" OR "functional limitation" OR "physical disability" OR "physical limitation" OR "physical impairment" OR "activities of daily living" OR ADL OR IADL).

To further evaluate specificity, the search was restricted to title and abstract fields, yielding 21,236 results:

("Middle Aged"[MeSH] OR "middle aged adults"[Title/Abstract] OR "midlife"[Title/Abstract] OR "mid-life"[Title/Abstract] OR "middle-aged adults"[Title/Abstract] OR "middle-aged individuals"[Title/Abstract] OR "45-64"[Title/Abstract] OR "55-64"[Title/Abstract] OR "60-69"[Title/Abstract]) AND ("mobility limitation"[Title/Abstract] OR "mobility impairments"[Title/Abstract] OR "mobility disability"[Title/Abstract] OR "functional impairments"[Title/Abstract] OR "functional disability"[Title/Abstract] OR "functional limitation"[Title/Abstract] OR "physical disability"[Title/Abstract] OR "physical limitation"[Title/Abstract] OR "physical impairment"[Title/Abstract] OR "activities of daily living"[Title/Abstract] OR ADL[Title/Abstract] OR IADL [Title/Abstract])

Therefore, the final search strategy was refined by adding methodological and measurement-related terms, yielding 5,454 results:

("Middle Aged"[MeSH] OR "middle aged adults" OR "midlife" OR "mid-life" OR "middle-aged adults" OR "middle-aged individuals" OR "midlife" OR "mid-life" OR "45-64" OR "55-64" OR "60-69") AND ("mobility limitation" OR "mobility impairments" OR "mobility disability" OR "functional impairments" OR "functional disability" OR "functional limitation" OR "physical disability" OR "physical limitation" OR "physical impairment" OR "activities of daily living" OR ADL OR IADL) AND (prevalence OR incidence) AND ("cohort" OR "cross-sectional" OR "wave"))

**Embase**

The initial broad search yielded 30,239 results using the following query:

('middle aged'/exp OR 'middle aged adults' OR 'middle-aged adults' OR 'middle aged individuals' OR 'middle-aged individuals' OR 'midlife' OR 'mid-life' OR '45-64' OR '55-64' OR '60-69') AND ('mobility limitation'/exp OR 'mobility disability' OR 'mobility impairments' OR 'functional limitation' OR 'functional disability' OR 'functional impairments' OR 'physical disability' OR 'physical limitations' OR 'physical impairments' OR 'activities of daily living'/exp OR adl OR iadl)

To further evaluate specificity, the search was restricted to title and abstract fields, yielding 471 results:

('middle aged adults':ti,ab OR 'middle-aged adults':ti,ab OR 'middle aged individuals':ti,ab OR 'middle-aged individuals':ti,ab OR 'midlife':ti,ab OR 'mid-life':ti,ab OR 'mid-age':ti,ab OR '45-64':ti,ab OR '55-64':ti,ab OR '60-69':ti,ab) AND ('mobility limitation':ti,ab OR 'mobility disability':ti,ab OR 'mobility impairments':ti,ab OR 'functional limitation':ti,ab OR 'functional disability':ti,ab OR 'functional impairments':ti,ab OR 'physical disability':ti,ab OR 'physical limitations':ti,ab OR 'physical impairments':ti,ab OR 'activities of daily living':ti,ab OR adl:ti,ab OR iadl:ti,ab)

The final search strategy incorporated epidemiological and study design terms, yielding 1,867 results (As this broader approach yielded a larger number of records, it was selected as the final search strategy):

('middle aged'/exp OR 'middle aged adults' OR 'middle-aged adults' OR 'middle aged individuals' OR 'middle-aged individuals' OR 'midlife' OR 'mid-life' OR '45-64' OR '55-64' OR '60-69') AND ('mobility limitation'/exp OR 'mobility disability' OR 'mobility impairments' OR 'functional limitation' OR 'functional disability' OR 'functional impairments' OR 'physical disability' OR 'physical limitations' OR 'physical impairments' OR 'activities of daily living'/exp OR adl OR iadl) AND (prevalence OR incidence) AND ('cohort study'/exp OR 'cross-sectional study'/exp OR ‘wave’)

**Web of Science**

The search yielded 1,527 results using the following query:

("middle aged adults" OR "middle-aged adults" OR "middle aged individuals" OR "middle-aged individuals" OR "midlife" OR "mid-life" OR "mid-age" OR "45-64" OR "55-64" OR "60-69") AND ("mobility limitation" OR "mobility disability" OR "mobility impairment" OR "functional limitation" OR "functional disability" OR "functional impairment" OR "physical disability" OR "physical limitation" OR "physical impairment" OR "activities of daily living" OR "ADL" OR "IADL")

**Scopus**

The initial broad search was conducted across all available fields, yielding 17,934 results:

ALL ( ( "middle aged adults" OR "middle-aged adults" OR "middle aged individuals" OR "middle-aged individuals" OR "midlife" OR "mid-life" OR "mid-age" OR "45-64" OR "55-64" OR "60-69" ) AND ( "mobility limitation" OR "mobility disability" OR "mobility impairment" OR "functional limitation" OR "functional disability" OR "functional impairment" OR "physical disability" OR "physical limitation" OR "physical impairment" OR "activities of daily living" OR "ADL" OR "IADL" ) )

To improve specificity while maintaining sensitivity, the search was limited to title, abstract, and keywords, yielding 856 results:

TITLE-ABS-KEY ( ( "middle aged adults" OR "middle-aged adults" OR "middle aged individuals" OR "middle-aged individuals" OR "midlife" OR "mid-life" OR "mid-age" OR "45-64" OR "55-64" OR "60-69" ) AND ( "mobility limitation" OR "mobility disability" OR "mobility impairment" OR "functional limitation" OR "functional disability" OR "functional impairment" OR "physical disability" OR "physical limitation" OR "physical impairment" OR "activities of daily living" OR "ADL" OR "IADL" ))

**Supplementary 3a: Critical appraisal assessment using the Joanna Briggs Institute (JBI) Critical Appraisal Checklist for Studies Reporting Prevalence Data**

| **Author and year of publication** | **1. Was the sample frame appropriate to address**  **the target population?** | | **2. Were study participants sampled in an appropriate way?** | | **3. Was the sample size adequate?** | | **4. Were the study subjects and setting described in detail?** | | **5. Was the data analysis conducted with sufficient coverage of the identified sample?** | | **6. Were valid methods used for the identification of the condition?** | | **7. Was the condition measured in a standard, reliable way for all participants?** | | **8. Was there appropriate statistical analysis?** | | **9. Was the response rate adequate, and if not, was the low response rate managed appropriately?** | | **Total number of “yes”** | | **Final Consensus Score** |
| --- | --- | --- | --- | --- | --- | --- | --- | --- | --- | --- | --- | --- | --- | --- | --- | --- | --- | --- | --- | --- | --- |
|  | **1** | **2** | **1** | **2** | **1** | **2** | **1** | **2** | **1** | **2** | **1** | **2** | **1** | **2** | **1** | **2** | **1** | **2** | **1** | **1** |  |
| Iezzoni et al., 2001 | y | y | y | y | y | y | u | u | y | y | u | y | u | u | y | y | u | u | 5 | 6 | 6 (Moderate) |
| Martin et al., 2010 | y | y | y | y | y | y | u | u | y | y | u | y | y | y | y | y | n | u | 6 | 7 | 6 (Moderate) |
| Karvonen-Gutierrez & Ylitalo, 2013 | n | n | y | y | u | u | y | y | y | y | y | y | y | y | y | y | u | u | 6 | 6 | 6 (Moderate) |
| Choi et al., 2016 | y | u | y | y | y | y | u | u | u | u | u | y | y | y | y | y | Y | Y | 6 | 6 | 6 (Moderate) |
| Brown et al., 2017 | y | y | y | y | y | y | y | y | y | y | y | y | y | y | y | y | y | y | 9 | 9 | 9 (High) |
| Brown et al., 2019 | y | y | y | y | y | y | y | y | y | y | y | y | y | y | y | y | y | y | 9 | 9 | 9 (High) |
| Tipirneni et al, 2021 | y | y | y | y | y | y | u | y | y | y | u | y | y | y | y | y | u | u | 7 | 8 | 7 (High) |
| Okoro et al., 2018 | y | y | y | y | y | y | y | y | y | y | u | u | y | y | y | y | u | u | 7 | 7 | 7 (High) |
| Bowling et al., 2019 | y | y | y | y | y | y | y | y | y | y | y | y | y | y | y | y | y | y | 9 | 9 | 9 (High) |
| Griffith et al., 2017 | y | y | y | y | y | y | y | y | y | u | y | y | y | y | y | y | u | u | 8 | 7 | 7 (High) |
| Heimbuch et al. 2023 | y | y | y | y | y | y | y | y | y | y | u | n | y | y | y | y | y | y | 8 | 8 | 8 (High) |
| Melzer et al., 2005 | y | y | y | y | y | y | y | y | y | y | u | u | y | y | y | y | u | u | 7 | 7 | 7 (High) |
| Gardener et al., 2006 | y | y | y | y | y | y | y | y | y | y | u | u | y | y | y | y | u | u | 7 | 7 | 7 (High) |
| Burns et al., 2024 | y | y | y | y | y | y | u | u | y | y | u | u | Y | Y | y | y | u | u | 6 | 6 | 6 (Moderate) |
| Wang et al., 2021 | y | y | y | y | y | y | y | y | y | y | y | y | u | y | y | y | u | u | 7 | 8 | 8 (High) |
| Beller et al., 2023 | y | y | y | y | y | y | y | y | y | y | y | y | y | y | y | y | u | u | 7 | 8 | 7 (High) |
| Picavet & Hoeymans, 2002 | y | y | y | y | y | y | n | u | y | y | u | y | y | y | y | y | u | u | 6 | 7 | 6 (Moderate) |
| Almazán-Isla, 2014 | y | y | y | y | y | y | u | u | y | y | y | y | y | y | y | y | u | u | 7 | 7 | 7 (High) |
| Wilk et al., 2023 | y | y | y | y | y | y | y | y | u | u | y | y | y | y | y | y | y | y | 8 | 8 | 8 (High) |
| Yonamine et al., 2016 | y | y | y | y | y | y | y | y | y | y | y | y | y | u | y | y | u | y | 8 | 8 | 8 (High) |
| Boakye et al., 2024 | y | y | y | y | u | u | y | y | u | u | y | y | y | y | y | u | n | n | 6 | 5 | 6 (Moderate) |
| Wilunda et al., 2015 | u | u | y | y | u | u | u | u | y | y | y | y | y | y | y | y | u | u | 5 | 5 | 5 (Moderate) |
| Ai et al. 2024 | y | y | y | y | y | y | y | y | u | y | n | n | y | y | y | y | u | n | 6 | 7 | 6 (Moderate) |
| Nagarkar et al., 2020 | y | y | y | y | y | y | y | y | y | y | y | y | y | y | y | y | y | y | 9 | 9 | 9 (High) |
| Peltzer et al. 2021 | y | y | y | y | y | y | y | y | y | y | n | n | y | y | y | y | y | y | 8 | 8 | 8 (High) |
| Zimmer et al., 2014 | y | y | y | y | y | y | u | u | y | y | y | u | u | u | y | y | u | u | 6 | 5 | 6 (Moderate) |

Note: "1" and "2" indicate the independent assessments by Reviewer 1 and Reviewer 2, respectively.

An item would be scored “0” if it was answered n -“No” or u -“Unclear”; if it was answered y -“Yes”, then the item scored “1”. Methodological quality will be considered “low,” “moderate,” and “high” if three or less, four to six, and seven to nine criteria will be met, respectively.

**Supplementary 3b: Risk of bias assessment using the “Assessing risk of bias in prevalence studies: modification of an existing tool” instrument proposed by Hoy et al. (2012).**

|  | **External Validity** | | | | | | | | **Internal Validity** | | | | | | | | | | | | |  | | |  |
| --- | --- | --- | --- | --- | --- | --- | --- | --- | --- | --- | --- | --- | --- | --- | --- | --- | --- | --- | --- | --- | --- | --- | --- | --- | --- |
| **Author and year of publication** | **1. Was the**  **study’s target**  **population a**  **close**  **representation**  **of the national**  **population in**  **relation to**  **relevant**  **variables, e.g.**  **age, sex,**  **occupation?** | | **2. Was the sampling frame a true or close representation of the target population?** | | **3. Was some form of random selection used to select the sample, OR, was a census undertaken?** | | **4. Was the likelihood of non-response bias minimal?** | | **5. Were data**  **collected**  **directly from**  **the subjects**  **(as opposed to**  **a proxy)?** | | **6. Was an acceptable case definition used in the study?** | | **7. Was the study instrument that measured the parameter of interest (e.g. prevalence of low back pain) shown to have reliability and validity (if necessary)?** | | **8. Was the same mode of data collection used for all subjects?** | | **9. Was the length of the shortest prevalence period for the parameter of interest appropriate?** | | **10. Were the numerator( s) and denominator(s) for the parameter of interest appropriate?** | | **Total “Yes”** | | | **Final Consensus Score (Low, Moderate, High)** |  |
|  | **1** | **2** | **1** | **2** | **1** | **2** | **1** | **2** | **1** | **2** | **1** | **2** | **1** | **2** | **1** | **2** | **1** | **2** | **1** | **2** | **1** | | **2** |  |  |
| Iezzoni et al., 2001 | y | y | y | y | y | y | n | n | n | y | y | y | y | y | n | y | y | y | y | y | 7 | | 8 | 7 (Moderate) |  |
| Martin et al., 2010 | y | y | y | y | y | y | n | n | y | n | y | y | y | y | y | y | y | y | y | y | 9 | | 8 | 8 (Low) |  |
| Karvonen-Gutierrez & Ylitalo, 2013 | n | n | n | n | y | y | n | n | y | y | y | y | y | y | y | y | y | y | y | y | 7 | | 7 | 7 (Moderate) |  |
| Choi et al., 2016 | y | y | y | y | y | y | n | n | n | y | y | y | y | y | n | n | y | y | y | y | 7 | | 8 | 7 (Moderate) |  |
| Brown et al., 2017 | y | y | y | y | y | y | n | n | y | y | y | y | y | y | n | n | y | y | y | y | 8 | | 8 | 8 (Low) |  |
| Brown et al., 2019 | y | y | y | y | y | y | n | n | y | y | y | y | y | y | y | n | y | y | y | y | 9 | | 8 | 8 (Low) |  |
| Tipirneni et al, 2021 | y | y | y | y | y | y | n | n | y | y | y | y | y | y | y | y | y | y | y | y | 9 | | 9 | 9 (Low) |  |
| Okoro et al., 2018 | y | y | y | y | y | y | n | n | y | y | y | y | y | y | y | y | y | y | y | y | 9 | | 9 | 9 (Low) |  |
| Bowling et al., 2019 | y | y | y | y | y | y | n | n | y | y | y | y | y | y | y | y | y | y | y | y | 9 | | 9 | 9 (Low) |  |
| Griffith et al., 2017 | y | y | y | y | y | y | y | y | y | y | y | y | y | y | y | y | y | y | y | y | 10 | | 10 | 10 (Low) |  |
| Heimbuch et al. 2023 | y | y | y | y | y | y | n | n | y | y | n | u | u | y | y | y | y | y | y | y | 7 | | 8 | 7 (Moderate) |  |
| Melzer et al., 2005 | y | y | y | y | y | y | n | n | n | n | y | y | y | y | y | n | y | y | y | y | 8 | | 7 | 7 (Moderate) |  |
| Gardener et al., 2006 | y | y | y | y | y | y | n | n | n | y | y | y | y | y | n | n | y | y | y | y | 7 | | 8 | 7 (Moderate) |  |
| Burns et al., 2024 | y | y | y | y | y | y | n | n | n | y | y | y | y | y | n | n | y | y | y | y | 7 | | 8 | 7 (Moderate) |  |
| Wang et al., 2021 | y | y | y | y | y | y | n | n | y | y | y | y | y | y | y | n | y | y | y | y | 9 | | 8 | 8 (Low) |  |
| Beller et al., 2023 | y | y | y | y | y | y | n | n | y | y | y | y | y | y | y | y | y | y | y | y | 9 | | 9 | 9 (Low) |  |
| Picavet & Hoeymans, 2002 | y | y | y | y | y | y | n | n | y | y | y | y | y | y | y | n | y | y | y | y | 9 | | 8 | 8 (Low) |  |
| Almazán-Isla, 2014 | n | n | y | y | y | y | y | y | y | y | y | y | y | y | y | n | y | y | y | y | 9 | | 8 | 8 (Low) |  |
| Wilk et al., 2023 | y | y | y | y | y | y | n | n | y | y | y | y | y | y | y | y | y | y | y | y | 9 | | 9 | 9 (Low) |  |
| Yonamine et al., 2016 | n | n | y | y | y | y | y | y | y | y | y | y | y | y | y | y | y | y | y | y | 9 | | 9 | 9 (Low) |  |
| Boakye et al., 2024 | y | y | u | n | y | y | n | n | y | u | y | y | y | y | y | y | y | y | y | y | 8 | | 7 | 7 (Moderate) |  |
| Wilunda et al., 2015 | n | n | y | y | y | y | n | n | y | y | y | y | y | y | y | y | y | y | y | y | 8 | | 8 | 8 (Low) |  |
| Ai et al. 2024 | y | y | y | y | y | y | n | n | y | y | u | u | u | n | y | y | y | y | y | y | 7 | | 7 | 7 (Moderate) |  |
| Nagarkar et al., 2020 | n | n | n | y | y | y | n | n | y | y | y | y | y | y | y | y | y | y | y | y | 7 | | 8 | 7 (Moderate) |  |
| Peltzer et al. 2021 | y | y | y | y | y | y | y | y | y | y | n | u | n | n | y | y | y | y | y | y | 7 | | 8 | 8 (Low) |  |
| Zimmer et al., 2014 | y | y | y | y | y | y | y | y | n | y | y | y | y | y | n | n | y | y | y | y | 8 | | 9 | 9 (Low) |  |

Note: The columns labeled "1" and "2" in the first row refer to the independent assessments conducted by Reviewer 1 and Reviewer 2, respectively. An item would be scored “0” if it was answered n -“No” (indicating a high risk of bias) ; if it was answered y -“Yes”(indicating a low risk of bias). Items for which insufficient information was available were also scored 'no'. Studies were categorized as 'High' risk of bias (i.e., if only 0-3 items were responded with yes, having an important impact on our confidence in the estimate, and will likely change the estimate), 'Moderate' (if 4-7 items were responded with yes), and 'Low' (if 8 or more items were responded with yes, indicating that further research is very unlikely to change our confidence in the estimate).

**Supplementary 4: GRADE evidence profile for functional and mobility limitations and associated health conditions**

| Outcome | Study design | N studies;  N participants | Quality of evidence assessment | | | | | | Summary of findings |
| --- | --- | --- | --- | --- | --- | --- | --- | --- | --- |
|  |  |  | Risk of bias | Inconsistency | Indirectness | Imprecision | Other considerations | Level of  evidence |  |
| **Primary Outcomes** | | | | | | | | | |
| Prevalence of Functional Limitations | Observational; cross-sectional and longitudinal studies | 17 studies; >600,000 participants | Not serious | Serious^1^ | Not serious | Not serious | None | ⨁⨁⨁◯ Moderate | ADL/IADL estimates ranged from 1.0% to 29.3%; one study reported estimates above 30%, (49.3%). |
| Prevalence of mobility limitations | Observational; cross-sectional and longitudinal studies | 16 studies; >390,000 participant | Not serious | Serious^2^ | Not serious | Not serious | None | ⨁⨁⨁◯ Moderate | Mobility limitation estimates ranged from 4.0% to 66.2%; estimates exceeded 40% in several settings. |
| Incidence of functional limitations | Observational; prospective longitudinal cohort studies | 3 studies; 18,655 participants | Not serious | Serious^3^ | Not serious | Serious^4^ | None | ⨁⨁◯◯ Low | ADL incidence ranged from 12.4% over 7 years to 19.8%-22.0% by age 64; IADL incidence ranged from 15.5% to 19.0% by age 64. |
| **Secondary Outcomes** | | | | | | | | | |
| Association of reported condition with functional and mobility limitations | Study design | N studies;  N participants | Quality of evidence assessment | | | | | | Summary of findings |
|  |  |  | Risk of bias | Inconsistency | Indirectness | Imprecision | Other considerations | Level of  evidence |  |
| Musculoskeletal pain/arthritis | Observational; cross-sectional and longitudinal studies | 5 studies; 32,020 participants | Serious^5^ | Serious^6^ | Serious^7^ | Serious^8^ | None | ⨁◯◯◯ Very low | ORs for mobility ranged from 1.71 for arthritis to 6.17 for lower-limb pain; PRs ranged from 2.21 to 3.13 across functional, mobility, BADL, and IADL outcomes; SHRs ranged from 1.58 to 2.02 for incident ADL/IADL impairment; HRs for incident ADL impairment were approximately 1.45-1.47. PAR was up to 38.0% for mobility limitations and 36.4% for IADL disability. |
| Obesity/overweight | Observational; cross-sectional and longitudinal studies | 2 studies; 11,091 participants | Serious^5^ | Serious^6^ | Serious^7^ | Serious^8^ | None | ⨁◯◯◯ Very low | PRs for obesity ranged from 1.30 to 1.68 across functional, mobility, BADL, and IADL outcomes; SHRs for obesity were 1.69 for incident ADL impairment and 1.28 for incident IADL impairment. ORs, HRs, and PARs were not reported. |
| Diabetes | Observational; cross-sectional and longitudinal studies | 4 studies; 25,779 participants | Serious^5^ | Serious^6^ | Serious^7^ | Serious^8^ | None | ⨁◯◯◯ Very low | OR for mobility limitation was 1.55 and crossed the null; PRs ranged from 1.10 to 1.39 across functional, mobility, BADL, and IADL outcomes; SHRs were 1.45-1.47 for incident ADL/IADL impairment. PAR was 2.0% for mobility limitations and up to 19.0% for IADL disability. |
| Stroke | Observational; cross-sectional and longitudinal studies | 3 studies; 16,883 participants | Serious^5^ | Serious^6^ | Serious^7^ | Serious^8^ | None | ⨁◯◯◯ Very low | OR for mobility limitation was 2.36; PRs ranged from 1.20 to 1.62 across functional, mobility, BADL, and IADL outcomes, with the mobility estimate crossing the null; SHRs were 2.25 for both incident ADL and IADL impairment. PAR for mobility limitations was 2.0%. |
| Heart disease | Observational; cross-sectional and longitudinal studies | 4 studies; 25,146 participants | Serious^5^ | Serious^6^ | Serious^7^ | Serious^8^ | None | ⨁◯◯◯ Very low | ORs for mobility limitation were 2.48 for angina and 1.88 for heart attack; PRs ranged from 1.21 to 1.58 across functional, mobility, BADL, and IADL outcomes, with several confidence intervals crossing the null; HR for incident ADL impairment was 1.37. PAR reached 18.7% for ADL disability and 29.7% for IADL disability in selected midlife strata. |
| Hypertension | Observational; cross-sectional and longitudinal studies | 4 studies; 23,124 participants | Serious^5^ | Serious^6^ | Serious^7^ | Serious^8^ | None | ⨁◯◯◯ Very low | OR for mobility limitation was 1.12 and crossed the null; PRs were close to the null across functional, mobility, BADL, and IADL outcomes; SHRs were approximately 1.18 for incident ADL/IADL impairment; HRs for incident ADL impairment were approximately 1.18–1.21 in middle-aged adults. PAR for mobility limitations was approximately 3.0% and imprecise. |
| Depression | Observational; cross-sectional and longitudinal studies | 4 studies; 25,779 participants | Serious^5^ | Serious^6^ | Serious^7^ | Serious^8^ | None | ⨁◯◯◯ Very low | PRs for depression ranged from 1.37 to 1.91 across functional, mobility, BADL, and IADL outcomes. |
| Multimorbidity | Observational; cross-sectional and longitudinal studies | 4 studies; 25,146 participants | Serious^5^ | Serious^6^ | Serious^7^ | Serious^8^ | None | ⨁◯◯◯ Very low | ORs for mobility limitation increased from 2.31 for one disease to 2.85 for three or more diseases; PRs increased with number of conditions, reaching 4.54 for functional limitations and 8.70 for mobility limitations among those with five or more conditions; HRs for incident ADL impairment were approximately 1.37 for one chronic disease and 1.73–1.84 for two or more chronic diseases. PAR ranged from 12.0% to 15.0% for mobility limitations and from 24.0% to 56.5% for functional disability. |

Abbreviations and GRADE domains: GRADE, Grading of Recommendations Assessment, Development and Evaluation; ADL, activities of daily living; IADL, instrumental activities of daily living. Certainty of evidence was assessed across the following GRADE domains. Risk of bias refers to limitations in study design, sampling, measurement, missing data, or reporting that may bias the estimate. Inconsistency refers to important variation in estimates across studies, including variation related to populations, instruments, thresholds, outcome definitions, or settings. Indirectness refers to differences between the included evidence and the review question in terms of population, outcome, measurement approach, or setting. Imprecision refers to uncertainty due to few studies, small samples, sparse events, wide confidence intervals, limited outcome coverage, or absence of pooled estimates. Other considerations included publication or reporting bias and any additional issues not captured by the preceding domains. Because meta-analysis was not performed, publication bias was not assessed statistically; instead, judgments were based on whether there was evidence of selective publication, selective outcome reporting, or limited availability of studies for a given outcome. Certainty was rated as high, moderate, low, or very low. Downgrading was applied when concerns in one or more domains limited confidence in the estimate.

^1^ Downgraded for inconsistency because estimates varied substantially across ADL/IADL domains, instruments, thresholds, and age groups, and because broader activity-limitation measures yielded higher estimates than domain-specific ADL/IADL measures.

^2^ Downgraded due to substantial variation in estimates across mobility tasks, instruments, thresholds, age strata, sex, and country settings.

^3^ Downgraded because incidence estimates were based on different follow-up structures and outcome definitions.

^4^ Downgraded because evidence was limited to three studies, with no pooled estimate and limited coverage across functional domains.

^5^ Downgraded because the association evidence was derived from observational studies, including cross-sectional analyses, with potential residual confounding, reverse causation, and differences in covariate adjustment across studies.

^6^ Downgraded because studies differed substantially in the chronic conditions assessed, exposure definitions, outcome domains, and effect measures, including ORs, PRs, HRs/SHRs, and PARs.

^7^ Downgraded because the evidence combined related but distinct outcomes, including functional limitations, ADL/IADL disability, mobility limitations, and physical limitations, and did not always provide condition-specific estimates separately for function and mobility.

^8^ Downgraded because each specific condition-outcome association was generally supported by a small number of studies, with several estimates having wide confidence intervals, confidence intervals crossing the null, or limited outcome coverage.
